# Prioritising Recommendations for Conversion to Living Mode in the Australasian Bronchiolitis Guideline: A Modified Delphi Study

**DOI:** 10.1002/gin2.70065

**Published:** 2026-03-16

**Authors:** Kate Loveys, Franz E. Babl, Elizabeth Cotterell, Libby Haskell, Sharon O'Brien, Ed Oakley, Emma J. Tavender, Catherine L. Wilson, Jane Alsweiler, Simon S. Craig, Nigel W. Crawford, Dianne Crellin, Sonja Crone, Trevor Duke, Shane George, Anna Lithgow, Ken Peacock, Tomas Ratoni, Annie Smith, Rebecca Starkie, David Thomas, Alexandra Wallace, Michael Zhang, Meredith L. Borland, Stuart R. Dalziel, the Paediatric Research in Emergency Departments International Collaborative (PREDICT) Network

**Affiliations:** ^1^ Department of Paediatrics: Child and Youth Health School of Medicine, The University of Auckland Auckland New Zealand; ^2^ Departments of Paediatrics and Critical Care University of Melbourne Parkville Victoria Australia; ^3^ Emergency Department Royal Children's Hospital Parkville Victoria Australia; ^4^ Clinical Sciences Murdoch Children's Research Institute Parkville Victoria Australia; ^5^ Armidale Rural Referral Hospital Armidale New South Wales Australia; ^6^ School of Rural Medicine and Tablelands Clinical School University of New England Armidale New South Wales Australia; ^7^ Children's Emergency Department Starship Children's Hospital Auckland New Zealand; ^8^ Emergency Department Perth Children's Hospital Perth Western Australia Australia; ^9^ Division of Emergency Medicine, Anaesthesia and Pain Medicine, Medical School University of Western Australia Perth Western Australia Australia; ^10^ Institute for Paediatric Perioperative Excellence The University of Western Australia Perth Western Australia Australia; ^11^ Australian Catholic University Sydney New South Wales Australia; ^12^ Department of Paediatrics Monash University Clayton Victoria Australia; ^13^ Paediatric Emergency Department Monash Medical Centre, Monash Health Clayton Victoria Australia; ^14^ Royal Children's Hospital Melbourne Victoria Australia; ^15^ Murdoch Children's Research Institute Melbourne Victoria Australia; ^16^ Department of Paediatrics University of Melbourne Melbourne Victoria Australia; ^17^ Department of Nursing University of Melbourne Melbourne Victoria Australia; ^18^ Department of Paediatrics Rotorua Hospital Te Whatu Ora Lakes Rotorua New Zealand; ^19^ Gold Coast University Hospital Southport Queensland Australia; ^20^ School of Medicine and Dentistry Griffith University Southport Queensland Australia; ^21^ Child Health Research Centre The University of Queensland Brisbane Queensland Australia; ^22^ Department of Paediatrics The Royal Darwin Hospital Darwin Northern Territory Australia; ^23^ Department of General Medicine, Children's Hospital at Westmead Sydney Children's Hospitals Network Westmead New South Wales Australia; ^24^ Northern NSW Local Health District Lismore New South Wales Australia; ^25^ Southland Hospital Invercargill New Zealand; ^26^ Department of General Practice and Primary Care, Melbourne Medical School, Faculty of Medicine, Dentistry and Health Sciences University of Melbourne Melbourne Victoria Australia; ^27^ Department of General Medicine Women's and Children's Hospital Adelaide South Australia Australia; ^28^ Department of Paediatrics, Waikato Hospital Te Whatu Ora Waikato Health NZ Hamilton New Zealand; ^29^ Emergency Department, John Hunter Hospital New Lambton Heights New South Wales Australia; ^30^ Department of Surgery, School of Medicine The University of Auckland Auckland New Zealand

**Keywords:** bronchiolitis, clinical guideline, infant, modified Delphi, paediatrics

## Abstract

**Background:**

The Australasian Bronchiolitis Guideline (ABG) provides evidence‐based recommendations for managing one of the most frequent reasons for infant hospitalisation. As traditional guidelines age, they suffer from lack of relevance when new evidence is available but not incorporated. To address this limitation a guideline, or parts thereof, can be converted into a “living” mode, where new evidence is regularly incorporated into recommendations, enabling faster translation to practice. This study aimed to prioritise ABG recommendations for conversion to a living mode.

**Method:**

Members of the 2025 ABG Guideline Advisory Group and Guideline Development Committee were invited to participate in an online, modified Delphi study. Panellists ranked 38 recommendations as low, medium, or high priority for conversion to a living mode across two rounds. A consensus threshold of ≥ 75% agreement was used. Data collection ceased once consensus was reached on ≥ 4 recommendations for conversion.

**Results:**

Twenty‐five of 29 (86%) experts responded in round one and 20/25 (80%) in round two. Panel diversity was maintained throughout voting rounds. At round one, consensus was reached to convert three recommendations to a living format and 11/38 recommendations were excluded. At round two, one additional recommendation was prioritised for conversion and 16/24 were excluded. Consensus was not reached on seven recommendations.

**Conclusion:**

Four key management recommendations will be converted to a living mode: high‐flow therapy, combined glucocorticoid/adrenaline therapy, oxygen saturation targets, and chest X‐ray in intensive care settings. These recommendations will be regularly updated as new evidence becomes available, supporting a more rapid translation to clinical practice.

## Summary

- The Australasian Bronchiolitis Guideline (ABG) provides evidence‐based clinical guidance for managing of one of the most common reasons for infant hospitalisation in Aotearoa New Zealand and Australia.
- Living guidelines enable more frequent recommendation updates on topics of high importance to interest‐holders, supporting a faster translation of new evidence to clinical practice.
- An online, modified Delphi study involving the ABG Guideline Advisory Group and Guideline Development Committee prioritised four existing guideline recommendations for conversion to living mode.
- These recommendations will be updated more frequently as new evidence emerges, providing clinicians with increased certainty on best practice. The prioritisation of recommendations in living mode will be reviewed over time.

## 1 Introduction

Bronchiolitis is one of the most common reasons for infants to present or be admitted to the hospital [1]. It is a seasonal acute respiratory illness prompted by a viral infection, mostly with respiratory syncytial virus (RSV). For many infants, bronchiolitis is self‐resolving. However, a proportion of infants will develop a more severe illness that requires hospital management for respiratory or hydration support.

Despite being a very common condition, historically bronchiolitis management in hospitals has been affected by practice variation, including use of ineffective therapies and investigations [2, 3]. In response to this evidence, along with the lack of Australasian‐specific guidance, the Paediatric Research in Emergency Departments International Collaborative (PREDICT) developed the Australasian Bronchiolitis Guideline in 2016 [4, 5]. This guideline aimed to provide evidence‐based, clinical guidance for the management of bronchiolitis in Australasian hospitals. The guideline covered bronchiolitis diagnosis, investigations, and therapies in paediatric emergency department (ED) and ward settings. Implementation of the guideline was shown to improve evidence‐based bronchiolitis management in Australasian hospitals [6].

More recently, the Australasian Bronchiolitis Guideline was updated in 2025 to include new evidence and to expand the scope to cover respiratory syncytial virus (RSV) immunisation, SARS‐CoV‐2 co‐infection, and intensive care unit (ICU) management [7, 8]. The updated guideline produced 41 recommendations spanning 25 topics. The guideline recommendations and methodology are detailed elsewhere [7, 8], and independent commentaries discuss the key changes [9, 10].

The Guideline update identified several important areas of bronchiolitis management with uncertainty in the evidence base, due to limited or low‐quality evidence [7, 11]. Some of these recommendations may benefit from adaption to a ‘living’ guideline mode. Living guidelines are a relatively new methodological approach that came to prominence during the COVID‐19 pandemic [12]. Living guidelines consist of regularly updating individual recommendations as new evidence emerges [12, 13], ensuring that recommendations are kept up to date. They are best suited to clinical questions that are high‐priority for decision‐making, with uncertainty in the existing evidence base, for which new evidence is expected to emerge that may change the recommendation [13, 14]. Living guidelines enable a more rapid translation of new evidence to clinical practice.

Methodological guidance for conducting living guidelines, including prioritising topics, is relatively new and continues to develop as the living approach becomes more commonplace [12]. Formal prioritisation methods, such as Delphi surveys or qualitative interviews, may provide a more transparent and robust process for determining interest‐holder preferences for the scope of a living guideline, which may improve the utility and uptake of the guidance. Current guidance from both the Australian Living Evidence Collaboration (ALEC), and an international working group (consisting of members of ALEC, the National Institute for Health and Care Excellence [NICE], and the United States Grading of Recommendations, Assessment, Development, and Evaluations [US GRADE] Network) recommends that interest‐holder consensus informs which topics are included in a living guideline [13, 14]. This study aimed to prioritise recommendations for conversion to a living mode in the Australasian Bronchiolitis Guideline using a modified Delphi approach.

## 2 Methods

### 2.1 Study Design

An online, modified Delphi study was designed following RAND methodological guidance [15], and reported in accordance with the ‘Delphi studies in social and health sciences—recommendations for an interdisciplinary standardized reporting’ (DELPHISTAR) checklist [16] (Supporting Information S1: Appendix 1). The protocol was registered on the Open Science Framework (ID hp2kt) [17].

A Delphi approach was considered appropriate as it is one method that has been recommended for prioritising topics for living guidelines by ALEC [13], and an international working group (ALEC, NICE, US GRADE Network) [14]. Methods of the classic Delphi approach were used, including iterative voting, controlled feedback, and statistical group summaries [15]. Modifications to the classic Delphi method included the lack of an open‐ended first voting round, where panellists generate ideas. Instead, panellists ranked the existing guideline topics, which they had previously prioritised as important through a recent consensus meeting at the outset of the Australasian Bronchiolitis Guideline 2025 update, in their capacity as members of the Guideline Advisory Group (GAG) or Guideline Development Committee (GDC) [7, 8, 11]. This approach was taken because we were evaluating recommendations to convert to a living mode from an established guideline which had been recently updated (published within 1 year of the Delphi study), with all topics reviewed by panellists. Secondly, we cannot claim that panellists were fully anonymous as they were recruited in their capacity as members of the GAG or GDC, and were therefore aware of who else was invited to participate based on group membership. However, our procedures aimed to maintain anonymity among panellists, who were unaware of who had participated in the invited group or of their responses. The survey administrator needed to be aware of who had responded for the purposes of sending reminders to participate and survey invitations. Thirdly, items that met the consensus threshold (≥ 75% agreement) during a voting round were removed from subsequent voting rounds. Aside from these modifications, a classic Delphi method was followed.

### 2.2 Panel Selection and Recruitment

All 29 members of the GAG and GDC of the Australasian Bronchiolitis Guideline 2025 update were invited to participate in the study over email. The group was comprised of clinical and academic experts specialising in areas relevant to bronchiolitis management (e.g., paediatric emergency, general paediatrics, intensive care, general practice), and guideline development and implementation. The group was diverse in terms of profession (e.g., medical, nursing, academic researcher), geographic location, hospital type, ethnicity, and gender make‐up. The group was predominantly senior medical (*n* = 20) or nursing specialists (*n* = 4). Thirteen members were senior academics (Associate Professor level or higher). All panellists were located in Aotearoa New Zealand or Australia. The panel included individuals of Indigenous ethnicity and/or who work in communities with a high proportion of Indigenous peoples. There were no significant conflicts of interest. The detailed information for each committee member is reported elsewhere [8, 18]. Panellists drew on their clinical experience and knowledge of the evidence base from their recent involvement in the Guideline update. The GAG (*n* = 10) managed the development of the latest guideline update including the contributors and methodology, evidence review, recommendation development, interest‐holder consultation, conflicts of interest, publication and dissemination. The GDC (*n* = 19) voted to determine the guideline scope, reviewed the evidence profile per topic, voted to finalise the recommendations, and reviewed the guideline publications.

To reduce attrition and maintain panel diversity, panellists were given three weeks to respond to each voting round with up to two personalised email reminders sent to those who had yet to participate in the round. Only panellists who had completed the first voting round were invited to participate in the second round. The survey rounds ran from 30/07/2025 to 21/08/2025 (round one), and 26/08/2025 to 16/09/2025 (round two).

### 2.3 Survey Content and Procedures

The panellists were asked to rank 38 of 41 existing guideline recommendations as high, medium, or low priority for conversion to a living mode, using a drop‐down menu within an online survey. The three recommendations on RSV immunisation were prospectively excluded from ranking, as the decision to use RSV vaccines or monoclonal antibodies (mAb) is made at a state or country level, rather than at an individual patient or hospital level. Moreover, at the time of the research, one of the two countries that this bi‐national guideline covers did not publicly fund RSV immunisation, therefore regular updates to clinical guidance on providing RSV immunisation would not be applicable to a proportion of the guideline end‐users at this time. The survey was hosted using Research Electronic Data Capture (REDCap), a secure web‐based platform [19, 20]. The survey was developed and pilot tested by the guideline co‐chairs (MLB, SRD), and methodologist (KL) from the Australasian Bronchiolitis Guideline 2025 update [11]. The Guideline co‐chairs are paediatric emergency medicine specialists and academics. External statistical consultation was not sought for the methodology.

**Figure 1:**
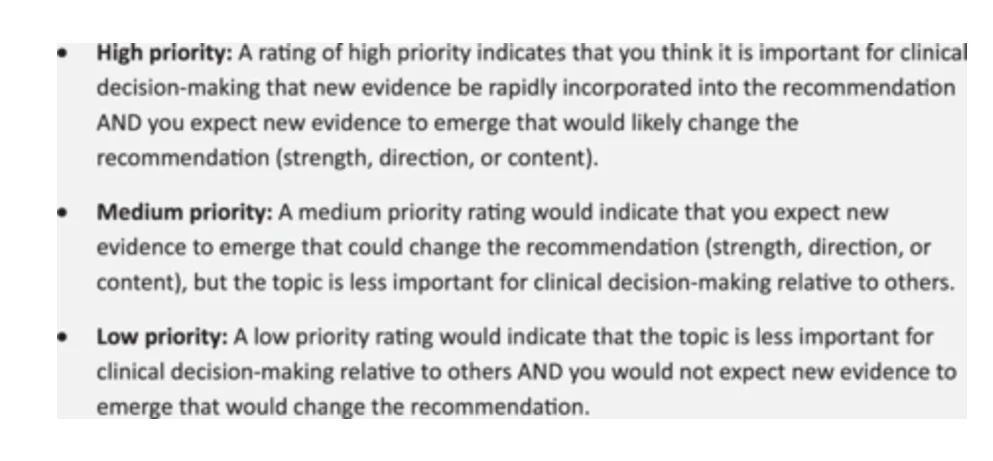
Definitions for high, medium, and low priority ranking categories. Definitions were informed by Australian Living Evidence Collaboration guidance [13].

Before completing round one, panellists were given a guidance document with information about living guidelines and how to prioritise recommendations, based on ALEC guidance [13]. The guidance stated that in order to be suitable for a living approach, the topic must be of high importance for clinical decision‐making (e.g., due to controversy or variation in clinical practice, emerging diseases, new therapies), and new evidence is expected to emerge that is likely to change the recommendation (e.g., studies are upcoming and feasible, and there is uncertainty in the evidence base). When ranking each recommendation, panellists were additionally informed of its Population, Intervention, Comparison, Outcome (PICO) question, recommendation text, recommendation strength, and evidence quality from the 2025 Guideline (detailed elsewhere [7, 11, 18]). The definitions for each ranking category (high, medium, low) were repeated for each item to encourage reliable responding (Figure 1 and Supporting Information S2 and S3: Appendices 2 and 3). At the end of the survey, panellists were asked to provide further written feedback on which recommendations should be converted to a living mode. Requests for modifications to the PICO questions from the 2025 guideline update were not explicitly sought due to the recency with which they were updated.

The consensus threshold for a recommendation to be converted to a living mode was ≥ 75% of votes at high and/or medium priority, or ≥ 75% of votes at low priority for exclusion. Round two only included recommendations for which the consensus threshold was not reached in round one. At the outset of round two, panellists were informed which recommendations were included for conversion to a living mode and excluded in the first round. Controlled feedback was given in the form of anonymous, aggregated summaries of the frequency and proportion of votes per ranking category for each recommendation in the first round, presented in bar charts with written summaries. All panellists received the same information. This summary allowed panellists to evaluate whether they or others held atypical opinions relative to the group. Panellists were asked to use the feedback to consider whether to change their vote. Panellists were not asked to justify their ranking of each recommendation in order to reduce participant burden. However, the open‐ended qualitative item at the end of the survey allowed panellists the opportunity to provide further comments. Appendices 2 and 3 present the surveys from each round.

The criteria for discontinuation of data collection were the first to occur of: (1) at least four recommendations reaching the consensus threshold for conversion to a living mode; or (2) three voting rounds. It was prospectively determined that four to six recommendations could be converted to a living mode based on the Guideline's resources. The limit of three voting rounds was used to avoid panel fatigue and forced consensus [15].

### 2.4 Data Analysis

As the data were categorical, frequency counts and proportions of votes received per ranking category for each recommendation and voting round were reported. The recommendations were categorised into ‘convert to a living mode,’ ‘exclude,’ and ‘consensus not reached,’ based on whether the consensus threshold was met during the last voting round. If more than the maximum of six recommendations had met the consensus threshold for conversion to a living mode, the first six recommendations with the greatest proportion of high‐priority votes would have been prioritised for conversion. There were no missing data. The ratings of all panellists were given equal weighting, and no subgroup analyses were performed.

Qualitative data were analysed by one reviewer using conventional content analysis to derive key themes, with review by a second researcher [21]. There were insufficient qualitative data (*n* = 2 comments) to appropriately triangulate qualitative and quantitative findings [22]; however, the complementarity of the findings was reported.

## 3 Results

**Figure 2:**
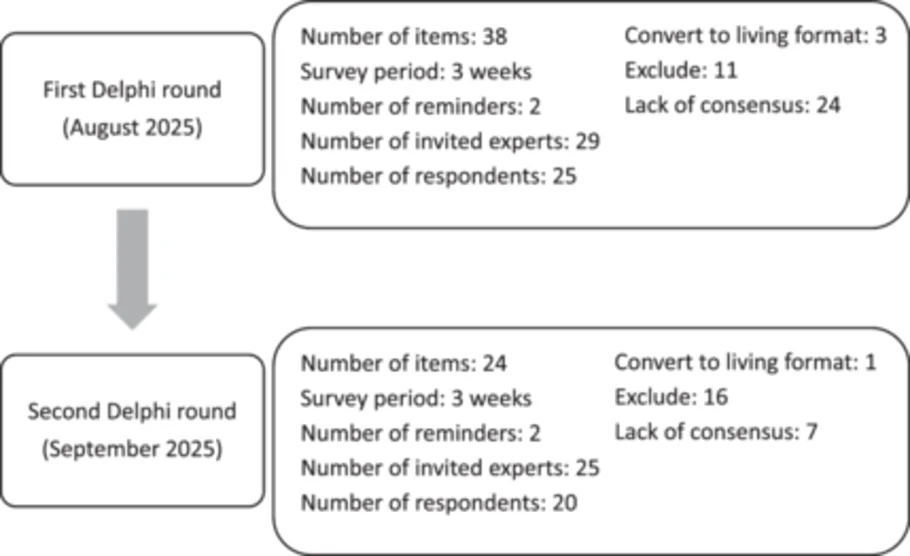
Delphi participant flow and process.

Of the 29 experts invited to complete the survey, 25 (86%) completed the first round. Twenty of 25 (80.0%) invited experts completed the second round. Table 1 presents the panel characteristics at each survey round. Overall, there was broad representation from different types of professions, areas of clinical expertise, and geographic locations in each survey round. The panellists were most often physicians (68%, round one; 65%, round two) or nurses (20%, round one; 20%, round two), with expertise in paediatric emergency medicine (40%, round one; 45%, round two), general paediatrics (28%, round one; 25%, round two), and other areas (e.g., paediatric intensive care, general practice, respiratory, immunology, neonatology). Most panellists were additionally experts in research, clinical epidemiology, and/or guideline development and implementation (84%, round one; 90%, round two). The panel was gender‐balanced in round one (56% female) and predominantly female in round two (70%). A flow chart depicts a summary of the study rounds, including attrition and the final classification of recommendations (Figure 2). There were no deviations from the intended approach.

**Table 1:** Panel characteristics.

Characteristics	Round one (*N* = 25) *n* (%)	Round two (*N* = 20) *n* (%)
Gender		
Female	14 (56)	14 (70)
Profession		
Physician	17 (68)	13 (65)
Nurse/nurse practitioner	5 (20)	4 (20)
Academic researcher (non‐clinical)	3 (12)	3 (15)
Expertise^a^		
Research, clinical epidemiology, or guideline development and implementation	21 (84)	18 (90)
Senior academic^b^	13 (52)	9 (45)
Paediatric emergency medicine	10 (40)	9 (45)
General paediatrics (incl. regional)	7 (28)	5 (25)
Paediatric intensive care	2 (8)	1 (5)
Other^c^	4 (16)	3 (15)
Location		
Metropolitan	20 (80)	15 (75)
Regional/rural	5 (20)	5 (25)
Country		
Aotearoa New Zealand	7 (28)	7 (35)
Australia	18 (72)	13 (65)

^a^ Expertise subcategories total over 100% due to the dual expertise of some panellists.

^b^ Associate Professor level or higher.

^c^ Other included general practice (*n* = 1), neonatology (*n* = 1), paediatric immunology (*n* = 1); paediatric respiratory (*n* = 1).

Overall, 38 recommendations were evaluated across two survey rounds. A summary of the recommendations assessed, included, and excluded at each round are presented in Table 2. At the end of round one, consensus was reached to include three recommendations and exclude 11 recommendations. This left 24 recommendations without consensus, which were voted on in round two. In round two, consensus was reached to include one additional recommendation for conversion to a living mode and exclude 16 recommendations. At this stage, the stopping criteria were met. Consensus was not reached on seven recommendations. Overall, four recommendations met the consensus criteria for conversion to a living mode: high‐flow therapy, combined glucocorticoid/adrenaline therapy, oxygen saturation targets, and chest X‐ray (CXR) in infants with severe bronchiolitis requiring high dependency unit (HDU)/intensive care unit (ICU) care (Figure 3).

**Table 2:** Final prioritisation of each recommendation for conversion to a living format from two voting rounds.

Topic	PICO question	2025 recommendation	Round one (*N* = 25)		Round two (*N* = 20)		Final outcome
			Votes to convert	Votes to exclude	Votes to convert	Votes to exclude	
			*n* (%)	*n* (%)	*n* (%)	*n* (%)	
Physical examination and history	In infants presenting to hospital, what factors in history and physical examination contribute to a differential diagnosis of bronchiolitis?	Consider a diagnosis of bronchiolitis in an infant if they have an upper respiratory tract infection (rhinorrhoea/nasal congestion, and/or cough), followed by the onset of a lower respiratory tract infection with one or more of respiratory distress (tachypnoea and/or retractions), or presence of diffuse crackles and/or wheeze, with or without the presence of fever. Additional signs and symptoms can include feeding difficulties, vomiting, dehydration, hypoxaemia, lethargy, uncommonly (< 5%) diarrhoea, and rarely (< 2%) apnoea. Significant change from 2016 recommendation: yes	2 (8)	23 (92)	—	—	Excluded round 1
Risk factors for severe bronchiolitis	In infants presenting to hospital with bronchiolitis, what are the risk factors for admission or severe disease (e.g., prolonged length of hospital stay, ICU admission, and death)?	Clinicians should take into account the following risk factors for more serious illness when assessing and managing infants with bronchiolitis: Gestational age < 37 weeks.* Younger chronological age at presentation.* Prenatal and/or postnatal exposure to tobacco smoke.* Reduced breastfeeding exposure.* Faltering growth/slow weight gain (failure to thrive). Comorbidities including congenital heart disease, chronic lung disease, chronic neurological condition, congenital diaphragmatic hernia, trisomy 21, and other genetic disorders. Being an Indigenous infant.† Being an economically disadvantaged infant. Timing and severity of illness onset at hospital presentation.	11 (44)	14 (56)	4 (20)	16 (80)	Excluded round 2
		*Clinicians should judge these as risk factors on a continuous scale; with higher risk of poor outcomes associated with lower gestational age, lower chronological age, fewer days of breastfeeding exposure, and greater tobacco smoke exposure. †Indigenous status in itself is unlikely to confer risk but there remains a correlation in Australia and Aotearoa New Zealand with ethnicity and severe bronchiolitis outcomes, independent of socioeconomic status, potentially reflecting the ongoing impacts of colonisation, remote geographical isolation, and the institutional racism in our health systems. Significant change from 2016 recommendation: yes					
Chest Xray (CXR)	In infants presenting to hospital or hospitalised with bronchiolitis, does performing a CXR at the time of presentation or admission beneficially change medical management or clinically relevant endpoints?	Do not routinely use CXR in infants presenting or admitted to hospital with bronchiolitis. Significant change from 2016 recommendation: no	6 (24)	19 (76)	—	—	Excluded round 1
	In infants who have an unexpected deterioration with bronchiolitis, does performing a CXR beneficially change medical management or clinically relevant endpoints?	Consider CXR in infants with an unexpected deterioration* and/or a clinical course not consistent with bronchiolitis, including concerns regarding the presence of sepsis, pneumonic consolidation, pneumothorax, empyema, immunodeficiency, pleural effusion, or significant cardiac abnormalities. *Unexpected deterioration refers to an unexpected requirement for an escalation of care. Significant change from 2016 recommendation: not applicable (new topic)	16 (64)	9 (36)	13 (65)	7 (35)	Consensus not reached
	In infants severely unwell with bronchiolitis (HDU/ICU level care), does performing CXR beneficially change medical management or clinically relevant endpoints?	Consider CXR in infants presenting with bronchiolitis in high dependency/intensive care settings, where there is clinician diagnostic concern regarding possible sepsis, pneumonic consolidation, pneumothorax, empyema, immunodeficiency, pleural effusion or significant complication of other diseases (e.g., heart failure with congenital heart disease), in order to guide treatment options. Significant change from 2016 recommendation: not applicable (new topic)	17 (68)	8 (32)	16 (80)	4 (20)	Converted to living mode round 2
Laboratory testing	In infants presenting to hospital or hospitalised with bronchiolitis, does performing laboratory tests (blood and/or urine), at the time of presentation or admission, beneficially change medical management or clinically relevant end‐points?	Do not routinely use laboratory tests for infants presenting to hospital or hospitalised with bronchiolitis, including bacteriological testing of urine or blood. Consider glucose and/or sodium levels during assessment in infants with bronchiolitis and poor feeding, evidence of dehydration or altered mental state. Significant change from 2016 recommendation: yes	4 (16)	21 (84)	—	—	Excluded round 1
	In infants who have an unexpected deterioration with bronchiolitis, does performing laboratory tests (blood and/or urine) beneficially change medical management or clinically relevant end‐points?	Consider use of biomarkers (e.g., FBC, CRP, PCT), urine testing, and blood cultures for the diagnosis of serious bacterial co‐infection for infants with unexpected deterioration during hospitalisation with bronchiolitis. Significant change from 2016 recommendation: not applicable (new topic)	16 (64)	9 (36)	14 (70)	6 (30)	Consensus not reached
	In infants severely unwell with bronchiolitis (HDU/ICU level care), does performing laboratory tests (blood and/or urine) beneficially change medical management or clinically relevant end‐points?	Consider use of biomarkers (e.g., FBC, CRP, PCT), urine testing, and blood cultures for the diagnosis of serious bacterial co‐infection for infants being admitted to ICU with bronchiolitis. Significant change from 2016 recommendation: not applicable (new topic)	16 (64)	9 (36)	14 (70)	6 (30)	Consensus not reached
Virological testing	In infants presenting to hospital or hospitalised with bronchiolitis, does performing virological investigations beneficially change medical management or clinically relevant end‐points?	Do not routinely use viral testing in infants presenting to hospital or hospitalised with bronchiolitis, including testing undertaken solely for cohorting of patients. Significant change from 2016 recommendation: no	11 (44)	14 (56)	2 (10)	18 (90)	Excluded round 2
Bronchiolitis severity scoring systems	In infants presenting to hospital or hospitalised with bronchiolitis, does use of a bronchiolitis scoring system beneficially change medical management or clinically relevant end‐points?	Do not routinely use a formal bronchiolitis severity scoring system to predict need for admission or hospital length of stay in infants presenting or admitted to hospital with bronchiolitis. Significant change from 2016 recommendation: no	11 (44)	14 (56)	5 (25)	15 (75)	Excluded round 2
Criteria for safe discharge	In infants presenting to hospital or hospitalised with bronchiolitis, what criteria should be used for safe discharge?	Safe discharge from hospital (either from the ED or ward) for infants with bronchiolitis should take into account risk factors (R2), the distance of the family's residence from the hospital and their ability to return, parental health literacy, and the timing of the hospital presentation relative to the natural history of bronchiolitis (R1). Consider patients suitable for safe discharge from hospital when the following criteria are met: 1. Infant is clinically stable (defined as with mild to moderate stable respiratory effort). 2. For an infant who has not received oxygen/respiratory support, and/or with SpO_2_ ≥ 95%, there is no need to continue to observe for maintenance of oxygen saturations. The infant may be considered for discharge based on the criteria below. For an infant who has received oxygen/respiratory support, and/or with SpO_2_ < 94%, they should be observed for maintenance of oxygen saturations in air at the following levels for 3‐4 h, including a period of sleep: i. For infants aged ≥ 6 weeks with no underlying health conditions, for maintenance of SpO_2_ ≥ 90%. ii. For infants aged < 6 weeks, or infants aged < 12 months with an underlying health condition, for maintenance of SpO_2_ ≥ 92%. 3. All infants irrespective of presentation to ED or on inpatient ward should be maintaining adequate oral intake of fluids and feeds of at least ½ of usual volume with adequate output (> 1/2 of usual wet nappies). 4. Parents and/or caregivers should feel confident to manage the infant with bronchiolitis at home. 5. Parents and/or caregivers are educated and provided with written information on possible deterioration and when to return for healthcare review. 6. Social situation allows discharge to home. The following factors should be considered: social factors, the time of day, and suitable transport availability. 7. Arrange local follow‐up where appropriate. Significant change from 2016 recommendation: yes	13 (52)	12 (48)	9 (45)	11 (55)	Consensus not reached
Beta2 agonists	In infants presenting to hospital or hospitalised with bronchiolitis, does administration of beta2 agonists (nebulisation, aerosol, oral or IV) improve clinically relevant end‐points?	Do not use beta2 agonists in infants (< 12 months of age) presenting to hospital or hospitalised with bronchiolitis. Significant change from 2016 recommendation: no	9 (36)	16 (64)	2 (10)	18 (90)	Excluded round 2
	In infants presenting to hospital or hospitalised with bronchiolitis, with a personal or family history of atopy, does administration of beta2 agonists (nebulisation, aerosol, oral or IV) improve clinically relevant end‐points?	Do not use beta2 agonists in infants (< 12 months of age) presenting to hospital or hospitalised with bronchiolitis, with a personal or family history of atopy outside of a RCT. Significant change from 2016 recommendation: no	7 (28)	18 (72)	2 (10)	18 (90)	Excluded round 2
Combination therapy (glucocorticoids and adrenaline)	In infants presenting to hospital or hospitalised with bronchiolitis, does administration of the combination of systemic or local glucocorticoids (nebulisation, oral, IM, or IV) and adrenaline improve clinically relevant end‐points?	Do not routinely use a combination of systemic or local corticosteroids and adrenaline/epinephrine in infants presenting to or hospitalised with moderate bronchiolitis outside of the ICU setting. Consider using a combination of systemic or local corticosteroids and adrenaline/epinephrine in infants with severe bronchiolitis requiring ICU level care. Significant change from 2016 recommendation: yes	21 (84)	4 (16)	—	—	Converted to living mode round 1
Adrenaline/epinephrine	In infants presenting to hospital or hospitalised with bronchiolitis, does administration of adrenaline/epinephrine (nebulisation, MDI, IM, IV) improve clinically relevant end‐points?	Do not use adrenaline/epinephrine in infants presenting to hospital or hospitalised with bronchiolitis. Significant change from 2016 recommendation: no	11 (44)	14 (56)	2 (10)	18 (90)	Excluded round 2
Glucocorticoids	In infants presenting to hospital or hospitalised with bronchiolitis, does administration of systemic or local glucocorticoids (nebulisation, oral, IM, or IV) improve clinically relevant end‐points?	Do not use systemic or local glucocorticoids in infants with bronchiolitis.* *For guidance on the use of glucocorticoids when SARS‐CoV‐2 infection is present, refer to ‘SARS‐CoV‐2 treatment.’ Significant change from 2016 recommendation: no	9 (36)	16 (64)	3 (15)	17 (85)	Excluded round 2
	In infants presenting to hospital or hospitalised with bronchiolitis, with a positive response to beta2 agonists, does administration of systemic or local glucocorticoids (nebulisation, oral, IM, or IV) improve clinically relevant end‐points?	Do not use glucocorticoids for the routine treatment of infants with bronchiolitis with a positive response to beta2 agonists or other markers of a latter asthmatic phenotype outside of a RCT. Beta2 agonists should not be used in infants aged < 12 months. Significant change from 2016 recommendation: no	9 (36)	16 (64)	2 (10)	18 (90)	Excluded round 2
Hypertonic saline	In infants presenting to hospital or hospitalised with bronchiolitis, does administration of nebulised hypertonic saline improve clinically relevant end‐points?	Do not routinely use nebulised hypertonic saline in infants presenting to hospital or hospitalised with bronchiolitis outside of a RCT. Significant change from 2016 recommendation: no	7 (28)	18 (72)	2 (10)	18 (90)	Excluded round 2
Azithromycin	In infants presenting to hospital or hospitalised with bronchiolitis, does the use of azithromycin medication improve clinically relevant end‐points?	Do not routinely use azithromycin for treatment of bronchiolitis in infants admitted to hospital. Significant change from 2016 recommendation: no	8 (32)	17 (68)	4 (20)	16 (80)	Excluded round 2
Antibiotic medication	In infants presenting to hospital or hospitalised with bronchiolitis, does the use of antibiotic medication improve clinically relevant end‐points?	Do not routinely use antibiotics for the treatment of infants with bronchiolitis. Significant change from 2016 recommendation: no	3 (12)	22 (88)	—	—	Excluded round 1
	In infants presenting to hospital or hospitalised with bronchiolitis, does the use of antibiotic medication in infants who are at risk of developing bronchiectasis improve clinically relevant end‐points?	Do not routinely use antibiotics for the treatment of bronchiolitis in infants who are at risk of developing bronchiectasis (due to known risk factors such as virus type (e.g., Adenovirus), Indigenous ethnicity, socioeconomic disadvantage). Significant change from 2016 recommendation: no	12 (48)	13 (52)	3 (15)	17 (85)	Excluded round 2
Supplemental oxygen and saturation targets	In infants presenting to hospital or hospitalised with bronchiolitis, does administration of supplemental oxygen improve clinically relevant end‐points?	Consider the use of supplemental oxygen in the treatment of hypoxaemic* infants with bronchiolitis. *For definitions of hypoxaemic and target oxygen saturation levels, see ‘Oxygen saturation targets.’ Significant change from 2016 recommendation: no	9 (36)	16 (64)	4 (20)	16 (80)	Excluded round 2
	In infants presenting to hospital or hospitalised with bronchiolitis, what level of oxygen saturation should lead to commencement or discontinuation of supplemental oxygen to improve clinically relevant end‐points?	Consider the use of supplemental oxygen in infants with bronchiolitis if their oxygen saturation is: Persistently < 90% for infants aged ≥ 6 weeks; Persistently < 92% for infants aged < 6 weeks, or infants aged < 12 months with an underlying health condition. Significant change from 2016 recommendation: yes	21 (84)	4 (16)	—	—	Converted to living mode round 1
Continuous pulse oximetry	In infants hospitalised with bronchiolitis, does continuous monitoring of pulse oximetry beneficially change medical management or clinically relevant end‐points?	Do not routinely use continuous pulse oximetry for medical management of non‐hypoxaemic infants (SpO_2_ ≥ 90% for infants aged ≥ 6 weeks age, or SpO_2_ ≥ 92% for infants aged < 6 weeks age, or infants aged < 12 months with an underlying health condition), with bronchiolitis not receiving oxygen, or stable infants receiving low‐flow oxygen, who are not at risk of apnoea. Significant change from 2016 recommendation: no	12 (48)	13 (52)	5 (25)	15 (75)	Excluded round 2
High‐flow (HF) therapy	In infants hospitalised with bronchiolitis, does the use of high‐flow nasal cannula improve clinically relevant end‐points?	Do not routinely use HF therapy in infants with mild or moderate bronchiolitis who are not hypoxaemic.* Do not routinely use HF therapy as a first‐line therapy in infants with moderate bronchiolitis who are hypoxaemic.* Consider HF therapy in infants with bronchiolitis who are hypoxaemic,* and who have failed low flow oxygen. Consider HF therapy in infants with bronchiolitis with severe disease prior to CPAP. *For otherwise healthy infants aged ≥ 6 weeks: SpO_2_ persistently < 90%. For infants aged < 6 weeks, or infants aged < 12 months with an underlying health condition: SpO_2_ persistently < 92%. Significant change from 2016 recommendation: yes	25 (100)	0 (0)	—	—	Converted to living mode round 1
Continuous positive airway pressure (CPAP)	In infants hospitalised with bronchiolitis, does the use of CPAP improve clinically relevant end‐points?	Consider using CPAP therapy in infants with bronchiolitis and impending or severe respiratory failure, and/or with severe illness. Significant change from 2016 recommendation: no	15 (60)	10 (40)	8 (40)	12 (60)	Consensus not reached
Feeding during high‐flow therapy	In infants presenting to hospital or hospitalised with bronchiolitis and managed with high‐flow therapy, does the use of enteral nutrition (oral and non‐oral) impact clinically relevant end‐points?	Consider enteral feeding (NG or oral), if tolerated, in infants receiving high flow. Consider continuous NG feeding in infants receiving CPAP who are not judged at imminent risk of intubation. Significant change from 2016 recommendation: not applicable (new topic)	12 (48)	13 (52)	6 (30)	14 (70)	Consensus not reached
Non‐oral hydration	In infants presenting to hospital or hospitalised with bronchiolitis, does the use of non‐oral hydration improve clinically relevant end‐points?	Use supplemental hydration for infants with bronchiolitis who cannot maintain hydration orally. Significant change from 2016 recommendation: no	4 (16)	21 (84)	—	—	Excluded round 1
	In infants presenting to hospital or hospitalised with bronchiolitis, what forms of non‐oral hydration improve clinically relevant end‐points?	Use either NG or IV routes for non‐oral hydration in infants admitted to hospital with bronchiolitis requiring supplemental hydration. Consider NG as the preferred first method of non‐oral hydration in infants with moderate bronchiolitis requiring supplemental hydration. Consider either continuous or bolus methods of NG non‐oral hydration with oral rehydration solution, breast milk, or formula in infants admitted to hospital with bronchiolitis requiring an NG. Significant change from 2016 recommendation: yes	8 (32)	17 (68)	1 (5)	19 (95)	Excluded round 2
	In infants presenting to hospital or hospitalised with bronchiolitis, does limiting the volume of non‐oral hydration impact on clinically relevant end‐points?	Consider fluid restriction at 50‐75% of recommended maintenance due to the risk of fluid overload from SiADH, and hyponatremia in bronchiolitis. Monitor for signs of overhydration. Significant change from 2016 recommendation: yes	9 (36)	16 (64)	3 (15)	17 (85)	Excluded round 2
	In infants presenting to hospital or hospitalised with bronchiolitis, does the type of intravenous fluid impact clinically relevant end‐points?	Consider using either 0.9% sodium chloride (normal saline) with 5% glucose, or balanced fluid (e.g., Plasma‐lyte 148^TM^ or Hartmann's solution) with 5% glucose, for use as maintenance fluid in infants admitted to hospital with bronchiolitis requiring IV hydration. For younger infants aged up to 4 weeks corrected with bronchiolitis, consider 10% glucose, or monitoring of blood sugar levels if receiving 5% glucose. Significant change from 2016 recommendation: not applicable (new topic)	5 (20)	20 (80)	—	—	Excluded round 1
Chest physiotherapy	In infants hospitalised with bronchiolitis, does chest physiotherapy improve clinically relevant end‐points?	Do not routinely use chest physiotherapy in infants with bronchiolitis. Significant change from 2016 recommendation: no	2 (8)	23 (92)	—	—	Excluded round 1
Suctioning	In infants hospitalised with bronchiolitis, does suctioning of the nose or nasopharynx improve clinically relevant end‐points?	Do not routinely use nasal suction in the management of infants with bronchiolitis. Consider using superficial suctioning in infants who have respiratory distress or feeding difficulties due to upper airway secretions. Significant change from 2016 recommendation: no	5 (20)	20 (80)	—	—	Excluded round 1
	In infants hospitalised with bronchiolitis, does deep suctioning in comparison to superficial suctioning beneficially improve clinically relevant end‐points?	Do not routinely use deep nasal suctioning for the management of infants with bronchiolitis. Significant change from 2016 recommendation: no	4 (16)	21 (84)	—	—	Excluded round 1
Nasal saline	In infants hospitalised with bronchiolitis, does the use of nasal saline drops improve clinically relevant end‐points?	Do not routinely use nasal saline drops in the management of infants with bronchiolitis. Consider a trial of intermittent nasal saline drops at time of feeding in infants with reduced feeding. Significant change from 2016 recommendation:	6 (24)	19 (76)	—	—	Excluded round 1
Infection control practices	In infants presenting to hospital or hospitalised with bronchiolitis, do infection control practices improve clinically relevant end‐points?	Use hand hygiene practices for the management of infants with bronchiolitis. Consider multicomponent infection control practices for the management of infants with bronchiolitis. Consider cohorting of infants admitted to inpatient wards with bronchiolitis. Significant change from 2016 recommendation: yes	7 (28)	18 (72)	0 (0)	20 (100)	Excluded round 2
SARS‐CoV‐2 co‐infection and treatment	In infants presenting to hospital or hospitalised with bronchiolitis, to what extent does SARS‐CoV‐2 virus infection, or co‐infection, contribute to disease incidence or severity?	Do not routinely use SARS‐CoV‐2 status to stratify increased risk for deterioration in infants with bronchiolitis. SARS‐CoV‐2 infection or co‐infection does not appear to place infants at increased risk of severe outcome from bronchiolitis. Significant change from 2016 recommendation: not applicable (new topic)	6 (24)	19 (76)	—	—	Excluded round 1
	In infants presenting to hospital or hospitalised with bronchiolitis, who test positive for SARS‐CoV‐2, does use of therapies targeting the SARS‐CoV‐2 virus (steroids, antivirals) improve clinically relevant end‐points?	Consider use of dexamethasone in hypoxic patients presenting with bronchiolitis who are also positive for SARS‐CoV‐2 co‐infection. Consider use of remdesivir in immunosuppressed infants who are also positive for SARS‐CoV‐2 infection. Significant change from 2016 recommendation: not applicable (new topic)	12 (48)	13 (52)	9 (45)	11 (55)	Consensus not reached

*Note:* Further information regarding the original and updated recommendations is available in the Australasian Bronchiolitis Guideline: 2025 update publications [7, 11, 18]. Consensus threshold ≥ 75% agreement.

Abbreviations: CPAP = continuous positive airway pressure, CRP = C‐reactive protein, CXR = chest X‐ray, ED = emergency department, FBC = full blood count, HF = high flow, ICU = intensive care unit, IV = intravenous, NG = nasogastric, PCT = procalcitonin, RCT = randomised controlled trial, RSV = respiratory syncytial virus, SARS‐CoV‐2 = severe acute respiratory syndrome coronavirus, SiADH = syndrome of inappropriate antidiuretic hormone secretion, SpO_2_ = peripheral oxygen saturation, UTI = urinary tract infection, wGA = weeks' gestational age.

Figure 3Recommendations prioritised for conversion to a living mode in the Australasian Bronchiolitis Guideline. CXR = chest X‐ray; HDU = high dependency unit; ICU = intensive care unit; IM = intramuscular; IV = intravenous; RQ = research question; SpO_2_ = oxygen saturation. ^1^The GRADE Evidence‐to‐Recommendation framework was used to translate the evidence to recommendations; further details of the methodology and the evidence profiles for each recommendation are published elsewhere [8, 11, 18]. ^2^Setting: emergency department, paediatric ward, ICU. ^3^Setting: HDU/ICU only.

**Figure:**
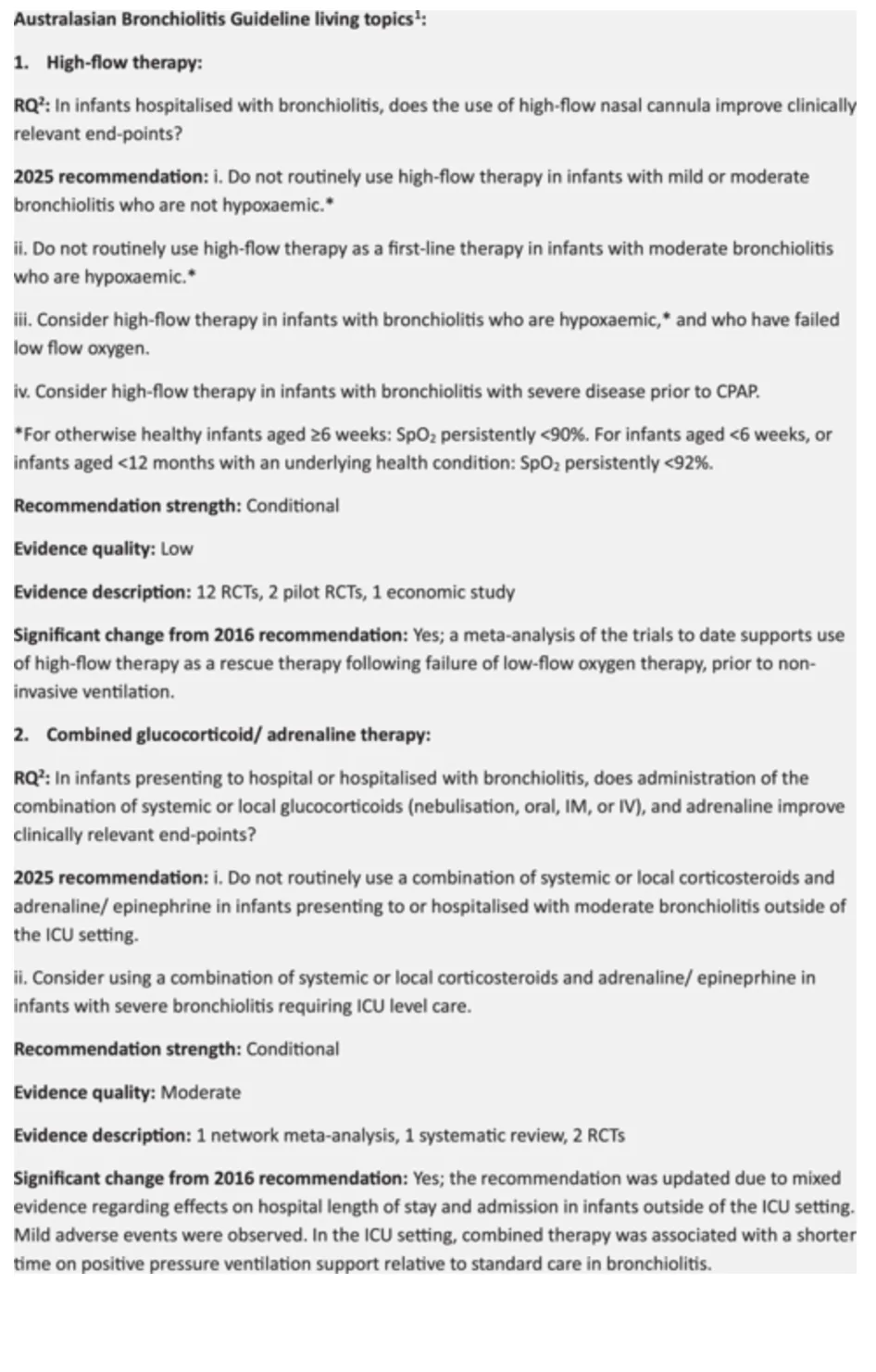

**Figure:**
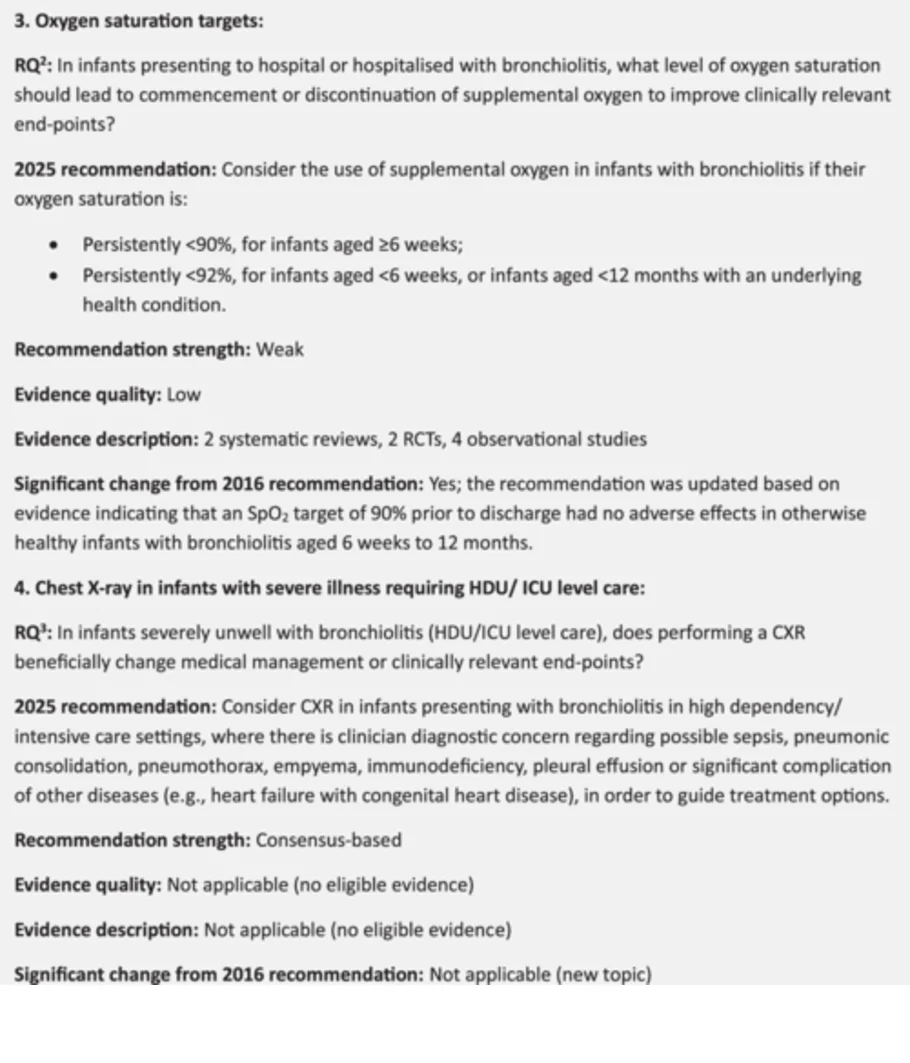

A total of two qualitative comments were received from 2/25 panellists, which complemented the quantitative data. Both comments expressed a theme of interest to prioritise additional recommendations: RSV immunisation (*n* = 1), general assessment (*n* = 1).

## 4 Discussion

Bronchiolitis is one of the most common reasons for infants to be hospitalised [1]. Yet bronchiolitis management in hospital has been historically affected by clinical practice variation, including use of ineffective therapies and investigations [2]. Bronchiolitis guidelines have been shown to improve evidence‐based management of the condition in hospital [6, 23, 24, 25]. However, for certain key areas of bronchiolitis management, the evidence to inform practice is limited or of low quality [11]. Living guidelines are a recent methodological advancement that are well‐suited to high‐priority clinical topics in which there is uncertainty in the evidence base, and new evidence is expected to inform the recommendation [12, 13, 14]. Living guidelines enable efficient translation of new evidence to clinical practice through regular evidence syntheses and recommendation updates [12]. In the context of bronchiolitis management, living recommendations would provide clinicians with increased certainty on best practice and may reduce practice variation. Indeed, in the 2025 update of the Australasian Bronchiolitis Guideline, 26 of 41 recommendations were found to have low to very low certainty evidence, and eight recommendations were consensus‐based due to a lack of eligible evidence (including in topics such as subgroups in which laboratory testing, CXR, or glucocorticoid use are warranted, intravenous fluid type and fluid restriction, treatment of SARS‐CoV‐2 co‐infection) [7, 11]. Although the updated recommendations were finalised with the support of a consensus majority (80% agreement), this was at times on the basis of uncertain or limited evidence, and a number of the recommendations may benefit from more recent updates as new evidence emerges to inform clinical practice. This study used a modified Delphi approach to prioritise recommendations for conversion to a living mode in the Australasian Bronchiolitis Guideline. This is the first bronchiolitis guideline to incorporate living recommendations, and the first study to prioritise recommendations from an existing bronchiolitis guideline for conversion to a living mode [7, 11].

In our study, recommendations on high‐flow therapy, combined glucocorticoid/adrenaline therapy, oxygen saturation targets, and CXR in infants with severe bronchiolitis with requirement for HDU/ICU level care were prioritised for conversion to a living mode by a diverse, local expert panel. High‐flow therapy was the most highly ranked recommendation for conversion to a living mode (100% agreement). Recent meta‐analyses on the efficacy of high‐flow compared to low flow oxygen have returned conflicting results in terms of effects on length of stay [26, 27], calling into question whether high‐flow should be used as a first‐line therapy. High‐flow remains an area with evidence of practice variation and low certainty evidence to inform best practice [18]. There are no other living guidelines for bronchiolitis, however high‐flow has been prioritised as an important management topic in other existing national guidelines for bronchiolitis [28, 29, 30].

Oxygen saturation targets and combined glucocorticoid/adrenaline therapy were equal second in their prioritisation for conversion to a living mode (84% agreement). Both topics have experienced recent shifts in recommended clinical practice, albeit based on limited evidence [7, 31]. Incoming trials may improve the certainty of the evidence base to confirm these shifts and possibly provide further evidence in relevant subgroups [32]. Oxygen saturation targets has been prioritised an important topic for patient management within most national bronchiolitis guidelines over the past two decades [33], whereas combined glucocorticoid/adrenaline therapy is a relatively new advancement [7, 28, 29]. Lastly, the role of CXR in infants with severe bronchiolitis requiring HDU/ICU care was prioritised (80% agreement). CXR has been the focus of de‐implementation interventions for infants with typical bronchiolitis through the Choosing Wisely campaign [34]. However, there is scant, very low quality evidence on the subgroups in which performing CXR may have clinical benefit [35]. Further evidence on this topic would provide clinicians with increased clarity. Routine CXR is commonly prioritised as a topic on which to provide clinicians with evidence‐based guidance in bronchiolitis guidelines [33]; fewer guidelines have made recommendations on CXR in HDU/ICU settings due to scope [7, 30].

Data collection ceased at the end of voting in round two, as the stopping criteria were met. This left seven guideline recommendations where consensus was not reached (Table 2). Two recommendations were found to have a split vote with < 60% agreement, two recommendations trended towards exclusion, and three recommendations trended towards conversion to a living mode but did not meet the consensus threshold (60‐74% agreement). In future prioritisation exercises, it is possible that some of these recommendations may be excluded or converted to a living format. Current methodological guidance suggests that the scope of living guidelines should be reviewed over time [14]. Recommendations may be retired from a living mode when a greater certainty of evidence is achieved and new evidence is unlikely to change the recommendation. Recommendations may also be transitioned from a static to a living format if prioritised due to high clinical need, evidence uncertainty, or the emergence of new evidence is likely to impact the recommendations. Indeed, retiring recommendations from a living mode may release resourcing for other recommendations to be converted to a living mode, although further topic prioritisation would need to occur. The very limited qualitative data that was received (*N* = 2 comments) asked about the possibility of including additional recommendations for ranking (e.g., RSV immunisation, overall assessment). Future prioritisation exercises could consider allowing panellists to input additional topics for ranking within the first round. This would enable us to capture new high priority clinical management topics that may have emerged since the last full guideline update, including innovations in care.

Living guidelines are a relatively new methodological approach that have become more commonplace following the COVID‐19 pandemic, as rigorous but regular evidence reviews and recommendation updates were required in response to a public health emergency [12]. Subsequently, living guidelines have been developed for a range of clinical areas (e.g., stroke [36], pregnancy and postnatal care [37, 38]). In tandem to the uptake of living guidelines, preliminary methodological guidance has been developed, which is in the process of being refined as further guidelines adopt and evaluate living methods [12, 14, 39, 40, 41, 42]. To date, there is limited guidance on how to select topics from an existing guideline for conversion to a living mode [14]. However, the Delphi method has been used to prioritise topics in other living guidelines [43, 44]. We found that an online, modified Delphi study was an appropriate method for our Guideline panel to prioritise recommendations for this purpose. It was a low‐cost, easy‐to‐use method for efficiently capturing consensus within a diverse expert panel, with a high response rate achieved. Several strategies may have improved the success of our method, including: (1) the provision of a brief, written document at the study outset with an introduction to living guidelines and factors to consider when prioritising recommendations for conversion to a living format, with content informed by current guidance [13, 14]; (2) provision of information from the latest guideline version (published within 1 year of the Delphi study) to support decision‐making on each recommendation, including the PICO question, recommendation text, recommendation strength, and evidence quality; (3) use of an online survey with a drop‐down menu to rank each recommendation, reducing the burden of participation; (4) pilot testing the survey; (5) provision of up to two personalised email reminders per survey round.

Alternative methods that have been recommended to prioritise topics for living guidelines have included qualitative interviews, surveys, or consensus meetings with interest‐holders (e.g., clinicians, consumers, evidence team, others [e.g., policymakers, healthcare funders]) [13, 14]. Qualitative interviews enable collection of rich data on interest‐holder perspectives. However, the data collection and analysis can be more time‐ and resource‐intensive relative to other methods. Consensus meetings allow for collection of quantitative and qualitative data in a less time‐ and resource‐intensive manner. However, the data collection may be negatively impacted by group dynamics, including conformity bias and social dominance by some participants. Surveys may enable more efficient data collection from a larger number of interest‐holders, as well as support a larger variety of quantitative assessment methods (e.g., Likert scale items). However, there is often less opportunity to collect rich qualitative data justifying responses. A number of living guidelines provide examples of how these alternative methods have been used for prioritising living guideline topics [45, 46, 47].

The UpPriority tool has been used to identify clinical questions for updating within existing guidelines [48]. This tool was not used in this study, as we had recently updated our guideline and it was not designed to prioritise topics for conversion to living recommendations [14, 49]. The UpPriority tool considers criteria such as the impact of outdated recommendations on safety, the availability of new relevant evidence, the context relevance of the clinical question, the methodological applicability of the clinical question, end‐user interest, and the impact on access to healthcare [49]. A guideline's clinical questions may meet the UpPriority criteria signalling a need for updating, without necessarily meeting the criteria for being appropriate for a living recommendation mode (i.e., the question is of high clinical importance, there is uncertainty in the evidence base, and new evidence is expected to emerge that could change the recommendation) [13, 14]. Living guideline developers could use the UpPriority tool to identify a shortlist of topics requiring an update within an outdated clinical practice guideline, followed by a separate exercise to prioritise the shortlisted topics for conversion to a living mode using recommended methods (e.g., Delphi surveys, consensus meetings, qualitative interviews) [13].

### 4.1 Strengths and Limitations

There were several strengths of our study. The Delphi approach is a widely accepted, structured method of evaluating expert consensus that avoids conformity bias and social dominance [50]. We maintained a high response rate (86%, round one; 80%, round two) and good panel diversity across voting rounds. Setting a maximum of three voting rounds reduced the likelihood of forced consensus and panel fatigue.

Use of an online survey may have helped with maintaining representation from diverse geographic locations, and regional and rural hospitals [50]. The broad spread of expertise, hospital types, and geographic locations represented within the guideline panel suggest that the prioritised recommendations are likely important across different types of interest‐holders. However, the results might not generalise to bronchiolitis guidelines in regions dissimilar to Aotearoa New Zealand and Australia, given there may be local variability in the perceived importance of different management topics or the availability of different therapies or investigations. Moreover, the findings of Delphi studies in general can have low replicability due to sensitivity to the panel make‐up [15]. Due to the small group size, it was not possible to conduct two concurrent panels to compare convergence in the findings.

An advantage of limiting participation to the GAG and GDC of the Australasian Bronchiolitis Guideline 2025 update was that feedback was sought from a diverse group of local experts with an up‐to‐date knowledge of the current evidence base, and the areas in which evidence is emerging. Experts were a mix of local clinicians and academics, with a broad spread of subspecialty and research expertise relevant to bronchiolitis, with representation from different hospital types (e.g., tertiary, regional/rural), in Aotearoa New Zealand and Australia. This provided representation of broad perspectives relevant to the guideline. However, the trade‐offs of this approach were that we had a smaller group from which to recruit panellists (*N* = 29), and we cannot claim full anonymity as panellists were aware of who else was invited to participate based on group membership. However, the use of an online survey, direct individual or blinded group‐level contact with respondents, and the provision of anonymised, aggregated feedback between survey rounds helped to maintain the anonymity of those who responded, and avoided social dominance and conformity bias that may be experienced in face‐to‐face group discussions [51]. Broader consultation from external clinicians was not sought because of access to a diverse group with an up‐to‐date knowledge of the evidence base for each clinical recommendation assessed in the Delphi survey. While we did not involve consumers directly in this work, the intended audience of the living recommendations is hospital clinicians and additional qualitative work is looking at consumer preferences for the management of this disease.

The panellists were not asked whether modifications should be made to any of the four PICO questions associated with the prioritised recommendations. This is because the PICO questions had recently been reviewed at the outset of the latest guideline update. During this process, three research questions were modified, three research questions were removed, 11 new research questions were added, and 27 research questions were kept from the 2016 guideline. Given that there have not been subsequent major changes in the field and the PICO questions were included as part of the survey exercise, we would expect that the current questions are acceptable for the four prioritised recommendations. However, question modifications should be assessed in the future. A further limitation is that our study did not explicitly ask panellists the reasons for their rankings, which could have allowed further understanding of atypical opinions should they have occurred. This survey adaption could be considered in future topic prioritisation surveys for living guidelines.

### 4.2 Future Research

The recommendations prioritised from this research will be the focus of living evidence syntheses and recommendation updates within the Australasian Bronchiolitis Guideline. The timing of the updates will be determined through an evidence mapping process considering factors such as the anticipated frequency, type, and size of new evidence per recommendation, and the implications for timelines, work plans, workforce capacity, and funding. In keeping with methodological guidance, ongoing prioritisation exercises will occur to ensure the highest priority bronchiolitis management topics are focused on, and recommendations that are no longer appropriate for a living mode are converted to static. The guideline recommendations on RSV immunisation that were excluded from prioritisation in this study could be included in future prioritisation exercises where appropriate. The scope of each PICO question will be reviewed alongside future topic prioritisation exercises to incorporate changes in the field or clinical priorities. The implementation of prevention strategies for RSV infection is likely to affect the prevalence of bronchiolitis given that RSV is the most common cause of bronchiolitis [52]. Future priorities will need to take this into consideration. Further research is needed to evaluate best practice methodology for both the development and implementation of living recommendations, and on how to most effectively engage Indigenous communities in the development of living guidelines. Some strategies could include: (1) ensuring Indigenous representation in guideline development committees, including clinicians, academics, and consumers, and clinicians who work in communities with a high proportion of Indigenous peoples; (2) ensuring Indigenous representation in consumer groups; (3) if consumer engagement involves qualitative research, ensuring that Indigenous consumers are purposively sampled and aim for equal group sizes by Indigenous and non‐Indigenous participants for equal explanatory power. Ensure that the Indigenous voice is not lost through involvement of Indigenous researchers during theme development and separate reporting of where the perspectives of Indigenous consumers vary; (4) seeking external consultation on the recommendations and their implementation from Indigenous experts, consumer groups, and related organisations.

## 5 Conclusion

Our modified Delphi study prioritised four recommendations for conversion to a living mode in the Australasian Bronchiolitis Guideline: high‐flow therapy, combined glucocorticoid/adrenaline therapy, oxygen saturation targets, and CXR in infants with severe bronchiolitis requiring HDU/ICU management. From the findings of this research, we will initiate living evidence syntheses and recommendation updates on these topics of critical importance to local interest‐holders. Adapting these topics to a living mode will enable rapid translation of related new evidence to clinical practice and provide clinicians with increased certainty on best practice in bronchiolitis management.

## Author Contributions

Conceptualisation: Kate Loveys, Meredith L. Borland, and Stuart R. Dalziel. Data curation: Kate Loveys. Formal analysis: Kate Loveys. Funding acquisition: Stuart R. Dalziel. Investigation: Kate Loveys, Meredith L Borland, and Stuart R. Dalziel. Methodology: Elizabeth Cotterell, Emma J. Tavender, Franz E. Babl, Kate Loveys, Meredith L Borland, and Stuart R. Dalziel. Project administration: Kate Loveys. Resources: Kate Loveys and Stuart R. Dalziel. Software: Kate Loveys. Supervision: Meredith L. Borland, and Stuart R. Dalziel. Validation: Kate Loveys, Meredith L. Borland, and Stuart R. Dalziel. Visualisation: Kate Loveys. Writing – original draft preparation: Kate Loveys. Writing – review and editing: all authors.

## Ethics Statement

This study was exempt from ethics approval as panellists participated in their capacity as members of a closed group, the Guideline Advisory Group and Guideline Development Committee of the Australasian Bronchiolitis Guideline 2025 update. Consent was implied by completion of the questionnaire.

## Conflicts of Interest

In accordance with ICMJE criteria, K.L., F.E.B., E.C., L.H., S.O., E.O., E.J.T., C.L.W., J.A., S.S.C., N.W.C., D.C., S.C., T.D., A.L., K.P., T.R., A.S., R.S., D.T., A.W., M.Z. have no conflicts of interest to declare. M.L.B. and S.R.D. have been provided with investigational product from Amphastar Pharmaceuticals Company, USA (epinephrine inhalers), and Trudell Medical, Canada (spacers), for a study of dexamethasone and epinephrine in bronchiolitis. S.G.'s institution has received funding from Fisher and Paykel Healthcare to support bronchiolitis research. None of the authors receive any personal financial benefits from industry sponsors. There were no industry sponsors for this study, nor did any of those listed have any role in the study's design, analysis, manuscript drafting, or decision to submit for publication.

## Supporting information

Appendix 1 ‐ DELPHISTAR reporting checklist.

Appendix 2 ‐ Delphi survey round one.

Appendix 3 ‐ Delphi survey round two.

## Data Availability

Data available on request from the authors.
