# Supplementary material for: Prioritising Recommendations for Conversion to Living Mode in the Australasian Bronchiolitis Guideline: A Modified Delphi Study
Source: Clin Public Health Guidel. 2026 Mar 16;3(2):e70065. doi: 10.1002/gin2.70065 (PMC13563326; doi:10.1002/gin2.70065)
Supplement: Supplementary file 1 [file GIN2-3-e70065-s001.pdf]

Prioritising topics from the Australasian Bronchiolitis Guideline for conversion to a living format

Background

Living guidelines are evidence-based guidelines that comprise one or more "living recommendations" that are continually updated as new information becomes available. We are planning to convert 4-6 high priority topics from the Australasian Bronchiolitis Guideline to a living format.

In this survey, we will ask you to rate each guideline topic as high, medium, or low priority for conversion to a living format. There are a total of 25 topics for you to rate. The survey should take about 20 minutes.

Please refer to the separate guidance document while completing the survey.

Should you have any questions or issues while completing the survey, please contact Kate (k.loveys@auckland.ac.nz), Stuart (s.dalziel@auckland.ac.nz), and Meredith (meredith.borland@health.wa.gov.au).

Your contact information:

Full name: ______________________________

Email: ______________________________

1. PHYSICAL EXAMINATION AND HISTORY

Please select whether you think that this guideline topic is of high, medium, or low priority for conversion to a living format.

Guidance for prioritising topics for conversion to a living format:

High priority: A rating of high priority indicates that you think it is important for clinical decision-making that new evidence be rapidly incorporated to the recommendation AND you expect new evidence to emerge that would likely change the recommendation (strength, direction, or content).

Medium priority: A medium priority rating would indicate that you expect new evidence that could change the recommendation (strength, direction, or content), but the topic is less important relative to others.

Low priority: A low priority rating would indicate that the topic is less important relative to others AND you would not expect new evidence to emerge that would change the recommendation.

Please rank the following topic:

PHYSICAL EXAMINATION AND HISTORY

Research question: In infants presenting to hospital, what factors in history and physical examination contribute to a differential diagnosis of bronchiolitis?

2025 recommendation: Consider a diagnosis of bronchiolitis in an infant if they have an upper respiratory tract infection (rhinorrhoea/ nasal congestion, and/or cough), followed by the onset of a lower respiratory tract infection with one or more of respiratory distress (tachypnoea and/or retractions), or presence of diffuse crackles and/or wheeze, with or without the presence of fever. Additional signs and symptoms can include feeding difficulties, vomiting, dehydration, hypoxaemia, lethargy, uncommonly (< 5%) diarrhoea, and rarely (< 2%) apnoea.

Quality of the 2025 evidence: very low

Strength of the 2025 recommendation: weak

What priority do you rate this topic for conversion to a living format?

- ◯ High priority
- ◯ Medium priority
- ◯ Low priority

2. RISK FACTORS

Please select whether you think that this guideline topic is of high, medium, or low priority for conversion to a living format.

Guidance for prioritising topics for conversion to a living format:

High priority: A rating of high priority indicates that you think it is important for clinical decision-making that new evidence be rapidly incorporated to the recommendation AND you expect new evidence to emerge that would likely change the recommendation (strength, direction, or content).

Medium priority: A medium priority rating would indicate that you expect new evidence that could change the recommendation (strength, direction, or content), but the topic is less important relative to others.

Low priority: A low priority rating would indicate that the topic is less important relative to others AND you would not expect new evidence to emerge that would change the recommendation.

Please rank the following topic:

RISK FACTORS

Research question: In infants presenting to hospital with bronchiolitis, what are the risk factors for admission or severe disease (e.g., prolonged length of hospital stay, ICU admission, and death)?

2025 recommendation: Clinicians should take into account the following risk factors for more serious illness when assessing and managing infants with bronchiolitis:

Gestational age < 37 weeks;* Younger chronological age at presentation;* Prenatal and/or postnatal exposure to tobacco smoke;* Reduced breastfeeding exposure;* Faltering growth/ slow weight gain (failure to thrive); Comorbidities including congenital heart disease, chronic lung disease, chronic neurological condition, congenital diaphragmatic hernia, trisomy 21, and other genetic disorders; Being an Indigenous infant†; Being an economically disadvantaged infant; Timing and severity of illness onset at hospital presentation. *Clinicians should judge these as risk factors on a continuous scale; with higher risk of poor outcomes associated with lower gestational age, lower chronological age, fewer days of breastfeeding exposure, and greater tobacco smoke exposure.

†Indigenous status in itself is unlikely to confer risk but there remains a correlation in Australia and Aotearoa New Zealand with ethnicity and severe bronchiolitis outcomes, independent of socioeconomic status, potentially reflecting the ongoing impacts of colonisation, remote geographical isolation, and the institutional racism in our health systems.

Quality of the 2025 evidence: moderate

Strength of the 2025 recommendation: strong

What priority do you rate this topic for conversion to a living format?	◯ High priority<br>◯ Medium priority<br>◯ Low priority

3. CHEST XRAY

Please select whether you think that this guideline topic is of high, medium, or low priority for conversion to a living format.

Guidance for prioritising topics for conversion to a living format:

High priority: A rating of high priority indicates that you think it is important for clinical decision-making that new evidence be rapidly incorporated to the recommendation AND you expect new evidence to emerge that would likely change the recommendation (strength, direction, or content).

Medium priority: A medium priority rating would indicate that you expect new evidence that could change the recommendation (strength, direction, or content), but the topic is less important relative to others.

Low priority: A low priority rating would indicate that the topic is less important relative to others AND you would not expect new evidence to emerge that would change the recommendation.

Please rank the following topic:

CHEST XRAY (CXR)

Research question: In infants presenting to hospital or hospitalised with bronchiolitis, does performing a CXR at the time of presentation or admission beneficially change medical management or clinically relevant endpoints?

2025 recommendation: Do not routinely use CXR in infants presenting or admitted to hospital with bronchiolitis.

Quality of the 2025 evidence: very low

Strength of the 2025 recommendation: conditional

What priority do you rate this topic for conversion to a living format?

◯ High priority
◯ Medium priority
◯ Low priority

Please rank the following topic:

CHEST XRAY (CXR)

Research question: In infants who have an unexpected deterioration with bronchiolitis, does performing a CXR beneficially change medical management or clinically relevant endpoints?

2025 recommendation: Consider CXR in infants with an unexpected deterioration* and/or a clinical course not consistent with bronchiolitis, including concerns regarding the presence of sepsis, pneumonic consolidation, pneumothorax, empyema, immunodeficiency, pleural effusion, or significant cardiac abnormalities.

*Unexpected deterioration refers to an unexpected requirement for an escalation of care.

Quality of the 2025 evidence: not applicable (no eligible evidence)

Strength of the 2025 recommendation: consensus-based

What priority do you rate this topic for conversion to a living format?

◯ High priority
◯ Medium priority
◯ Low priority

Please rank the following topic:

CHEST XRAY (CXR)

Research question: In infants severely unwell with bronchiolitis (HDU/ICU level care), does performing CXR beneficially change medical management or clinically relevant endpoints?

2025 recommendation: Consider CXR in infants presenting with bronchiolitis in high dependency/ intensive care settings, where there is clinician diagnostic concern regarding possible sepsis, pneumonic consolidation, pneumothorax, empyema, immunodeficiency, pleural effusion or significant complication of other diseases (e.g., heart failure with congenital heart disease), in order to guide treatment options.

Quality of the 2025 evidence: not applicable (no eligible evidence)

Strength of the 2025 recommendation: consensus-based

What priority do you rate this topic for conversion to a living format?	◯ High priority<br>◯ Medium priority<br>◯ Low priority

4. LABORATORY TESTING

Please select whether you think that this guideline topic is of high, medium, or low priority for conversion to a living format.

Guidance for prioritising topics for conversion to a living format:

High priority: A rating of high priority indicates that you think it is important for clinical decision-making that new evidence be rapidly incorporated to the recommendation AND you expect new evidence to emerge that would likely change the recommendation (strength, direction, or content).

Medium priority: A medium priority rating would indicate that you expect new evidence that could change the recommendation (strength, direction, or content), but the topic is less important relative to others.

Low priority: A low priority rating would indicate that the topic is less important relative to others AND you would not expect new evidence to emerge that would change the recommendation.

Please rank the following topic:

LABORATORY TESTING

Research question: In infants presenting to hospital or hospitalised with bronchiolitis, does performing laboratory tests (blood and/or urine), at the time of presentation or admission, beneficially change medical management or clinically relevant end-points?

2025 recommendation: i. Do not routinely use laboratory tests for infants presenting to hospital or hospitalised with bronchiolitis, including bacteriological testing of urine or blood.

ii. Consider glucose and/or sodium levels during assessment in infants with bronchiolitis and poor feeding, evidence of dehydration or altered mental state.

Quality of the 2025 evidence: very low

Strength of the 2025 recommendation: conditional

What priority do you rate this topic for conversion to a living format?

○ High priority
○ Medium priority
○ Low priority

Please rank the following topic:

LABORATORY TESTING

Research question: In infants who have an unexpected deterioration with bronchiolitis, does performing laboratory tests (blood and/or urine) beneficially change medical management or clinically relevant end-points?

2025 recommendation: Consider use of biomarkers (e.g., FBC, CRP, PCT), urine testing, and blood cultures for the diagnosis of serious bacterial co-infection for infants with unexpected deterioration during hospitalisation with bronchiolitis.

Quality of the 2025 evidence: not applicable (no eligible evidence)

Strength of the 2025 recommendation: consensus-based

What priority do you rate this topic for conversion to a living format?

○ High priority
○ Medium priority
○ Low priority

Please rank the following topic:

LABORATORY TESTING

Research question: In infants severely unwell with bronchiolitis (HDU/ ICU level care), does performing laboratory tests (blood and/or urine) beneficially change medical management or clinically relevant end-points?

2025 recommendation: Consider use of biomarkers (e.g., FBC, CRP, PCT) and blood cultures for diagnosis of serious bacterial co-infection for infants being admitted to ICU with bronchiolitis.

Quality of the 2025 evidence: very low

Strength of the 2025 recommendation: weak

What priority do you rate this topic for conversion to a living format?	◯ High priority<br>◯ Medium priority<br>◯ Low priority

5. VIROLOGICAL TESTING

Please select whether you think that this guideline topic is of high, medium, or low priority for conversion to a living format.

Guidance for prioritising topics for conversion to a living format:

High priority: A rating of high priority indicates that you think it is important for clinical decision-making that new evidence be rapidly incorporated to the recommendation AND you expect new evidence to emerge that would likely change the recommendation (strength, direction, or content).

Medium priority: A medium priority rating would indicate that you expect new evidence that could change the recommendation (strength, direction, or content), but the topic is less important relative to others.

Low priority: A low priority rating would indicate that the topic is less important relative to others AND you would not expect new evidence to emerge that would change the recommendation.

Please rank the following topic:

VIROLOGICAL TESTING

Research question: In infants presenting to hospital or hospitalised with bronchiolitis, does performing virological investigations beneficially change medical management or clinically relevant end-points?

2025 recommendation: Do not routinely use viral testing in infants presenting to hospital or hospitalised with bronchiolitis, including testing undertaken solely for cohorting of patients.

Quality of the 2025 evidence: very low

Strength of the 2025 recommendation: conditional

What priority do you rate this topic for conversion to a living format?

- ◯ High priority
- ◯ Medium priority
- ◯ Low priority

6. BRONCHIOLITIS SEVERITY SCORING SYSTEMS

Please select whether you think that this guideline topic is of high, medium, or low priority for conversion to a living format.

Guidance for prioritising topics for conversion to a living format:

High priority: A rating of high priority indicates that you think it is important for clinical decision-making that new evidence be rapidly incorporated to the recommendation AND you expect new evidence to emerge that would likely change the recommendation (strength, direction, or content).

Medium priority: A medium priority rating would indicate that you expect new evidence that could change the recommendation (strength, direction, or content), but the topic is less important relative to others.

Low priority: A low priority rating would indicate that the topic is less important relative to others AND you would not expect new evidence to emerge that would change the recommendation.

Please rank the following topic:

BRONCHIOLITIS SEVERITY SCORING SYSTEMS

Research question: In infants presenting to hospital or hospitalised with bronchiolitis, does use of a bronchiolitis scoring system beneficially change medical management or clinically relevant end-points?

2025 recommendation: Do not routinely use a formal bronchiolitis severity scoring system to predict need for admission or hospital length of stay in infants presenting or admitted to hospital with bronchiolitis.

Quality of the 2025 evidence: very low

Strength of the 2025 recommendation: weak

What priority do you rate this topic for conversion to a living format?

◯ High priority
◯ Medium priority
◯ Low priority

7. CRITERIA FOR SAFE DISCHARGE

Please select whether you think that this guideline topic is of high, medium, or low priority for conversion to a living format.

Guidance for prioritising topics for conversion to a living format:

High priority: A rating of high priority indicates that you think it is important for clinical decision-making that new evidence be rapidly incorporated to the recommendation AND you expect new evidence to emerge that would likely change the recommendation (strength, direction, or content).

Medium priority: A medium priority rating would indicate that you expect new evidence that could change the recommendation (strength, direction, or content), but the topic is less important relative to others.

Low priority: A low priority rating would indicate that the topic is less important relative to others AND you would not expect new evidence to emerge that would change the recommendation.

Please rank the following topic:

CRITERIA FOR SAFE DISCHARGE

Research question: In infants presenting to hospital or hospitalised with bronchiolitis, what criteria should be used for safe discharge?

2025 recommendation: Safe discharge from hospital (either from the ED or ward) for infants with bronchiolitis should take into account risk factors (R2), the distance of the family's residence from the hospital and their ability to return, parental health literacy, and the timing of the hospital presentation relative to the natural history of bronchiolitis (R1). Consider patients suitable for safe discharge from hospital when the following criteria are met:

1. Infant is clinically stable (defined as with mild to moderate stable respiratory effort).

2. For an infant who has not received oxygen/respiratory support and/or with $SpO_2 \geq 95\%$, there is no need to continue to observe for maintenance of oxygen saturations. The infant may be considered for discharge based on criteria below.

For an infant who has received oxygen/respiratory support and/or with $SpO_2 \leq 94\%$, they should be observed for maintenance of oxygen saturations in air at the following levels for 3-4 hours, including a period of sleep:
i. for infants aged ≥6 weeks with no underlying health conditions, for maintenance of $SpO_2 \geq 90\%$;
ii. for infants aged < 6 weeks, or infants aged < 12 months with an underlying health condition, for maintenance of $SpO_2 \geq 92\%$.

3. All Infants irrespective of presentation to ED or on inpatient ward should be maintaining adequate oral intake of fluids and feeds of at least 1/2 of usual volume with adequate output (>1/2 of usual wet nappies).

4. Parents and/or caregivers should feel confident to manage the infant with bronchiolitis at home.

5. Parents and/or caregivers are educated and provided with written information on possible deterioration and when to return for healthcare review.

6. Social situation allows discharge to home. The following factors should be considered: social factors, the time of day and suitable transport availability.

7. Arrange local follow-up where appropriate.

Quality of the 2025 evidence: very low

Strength of the 2025 recommendation: weak

What priority do you rate this topic for conversion to a living format?

- ◯ High priority
- ◯ Medium priority
- ◯ Low priority

8. BETA2 AGONISTS

Please select whether you think that this guideline topic is of high, medium, or low priority for conversion to a living format.

Guidance for prioritising topics for conversion to a living format:

High priority: A rating of high priority indicates that you think it is important for clinical decision-making that new evidence be rapidly incorporated to the recommendation AND you expect new evidence to emerge that would likely change the recommendation (strength, direction, or content).

Medium priority: A medium priority rating would indicate that you expect new evidence that could change the recommendation (strength, direction, or content), but the topic is less important relative to others.

Low priority: A low priority rating would indicate that the topic is less important relative to others AND you would not expect new evidence to emerge that would change the recommendation.

Please rank the following topic:

BETA2 AGONISTS

Research question: In infants presenting to hospital or hospitalised with bronchiolitis, does administration of beta2 agonists (nebulisation, aerosol, oral or IV) improve clinically relevant end-points?

2025 recommendation: Do not use beta2 agonists in infants (< 12 months of age) presenting to hospital or hospitalised with bronchiolitis.

Quality of the 2025 evidence: moderate

Strength of the 2025 recommendation: strong

What priority do you rate this topic for conversion to a living format?

- ◯ High priority
- ◯ Medium priority
- ◯ Low priority

Please rank the following topic:

BETA2 AGONISTS

Research question: In infants presenting to hospital or hospitalised with bronchiolitis, with a personal or family history of atopy, does administration of beta2 agonists (nebulisation, aerosol, oral or IV) improve clinically relevant end-points?

2025 recommendation: Do not use beta2 agonists in infants (< 12 months of age) presenting to hospital or hospitalised with bronchiolitis, with a personal or family history of atopy outside of a RCT.

Quality of the 2025 evidence: very low

Strength of the 2025 recommendation: strong

What priority do you rate this topic for conversion to a living format?

- ◯ High priority
- ◯ Medium priority
- ◯ Low priority

9. COMBINATION THERAPY (GLUCOCORTICOIDS AND ADRENALINE)

Please select whether you think that this guideline topic is of high, medium, or low priority for conversion to a living format.

Guidance for prioritising topics for conversion to a living format:

High priority: A rating of high priority indicates that you think it is important for clinical decision-making that new evidence be rapidly incorporated to the recommendation AND you expect new evidence to emerge that would likely change the recommendation (strength, direction, or content).

Medium priority: A medium priority rating would indicate that you expect new evidence that could change the recommendation (strength, direction, or content), but the topic is less important relative to others.

Low priority: A low priority rating would indicate that the topic is less important relative to others AND you would not expect new evidence to emerge that would change the recommendation.

Please rank the following topic:

COMBINATION THERAPY (GLUCOCORTICOIDS AND ADRENALINE)

Research question: In infants presenting to hospital or hospitalised with bronchiolitis, does administration of the combination of systemic or local glucocorticoids (nebulisation, oral, IM, or IV) and adrenaline improve clinically relevant end-points?

2025 recommendation: i. Do not routinely use a combination of systemic or local corticosteroids and adrenaline/epinephrine in infants presenting to or hospitalised with moderate bronchiolitis outside of the ICU setting.

ii. Consider using a combination of systemic or local corticosteroids and adrenaline/epinephrine in infants with severe bronchiolitis requiring ICU level care.

Quality of the 2025 evidence: moderate

Strength of the 2025 recommendation: conditional

What priority do you rate this topic for conversion to a living format?

- ◯ High priority
- ◯ Medium priority
- ◯ Low priority

10. ADRENALINE/ EPINEPHRINE

Please select whether you think that this guideline topic is of high, medium, or low priority for conversion to a living format.

Guidance for prioritising topics for conversion to a living format:

High priority: A rating of high priority indicates that you think it is important for clinical decision-making that new evidence be rapidly incorporated to the recommendation AND you expect new evidence to emerge that would likely change the recommendation (strength, direction, or content).

Medium priority: A medium priority rating would indicate that you expect new evidence that could change the recommendation (strength, direction, or content), but the topic is less important relative to others.

Low priority: A low priority rating would indicate that the topic is less important relative to others AND you would not expect new evidence to emerge that would change the recommendation.

Please rank the following topic:

ADRENALINE/ EPINEPHRINE

Research question: In infants presenting to hospital or hospitalised with bronchiolitis, does administration of adrenaline/ epinephrine (nebulisation, MDI, IM, IV) improve clinically relevant end-points?

2025 recommendation: Do not use adrenaline/ epinephrine in infants presenting to hospital or hospitalised with bronchiolitis.

Quality of the 2025 evidence: low

Strength of the 2025 recommendation: strong

What priority do you rate this topic for conversion to a living format?

- ◯ High priority
- ◯ Medium priority
- ◯ Low priority

11. GLUCOCORTICOIDS

Please select whether you think that this guideline topic is of high, medium, or low priority for conversion to a living format.

Guidance for prioritising topics for conversion to a living format:

High priority: A rating of high priority indicates that you think it is important for clinical decision-making that new evidence be rapidly incorporated to the recommendation AND you expect new evidence to emerge that would likely change the recommendation (strength, direction, or content).

Medium priority: A medium priority rating would indicate that you expect new evidence that could change the recommendation (strength, direction, or content), but the topic is less important relative to others.

Low priority: A low priority rating would indicate that the topic is less important relative to others AND you would not expect new evidence to emerge that would change the recommendation.

Please rank the following topic:

GLUCOCORTICOIDS

Research question: In infants presenting to hospital or hospitalised with bronchiolitis, does administration of systemic or local glucocorticoids (nebulisation, oral, IM, or IV) improve clinically relevant end-points?

2025 recommendation: Do not use systemic or local glucocorticoids in infants with bronchiolitis*.
*For guidance on the use of glucocorticoids when SARS-CoV-2 infection is present, refer to R22b 'SARS-CoV-2 treatment.'

Quality of the 2025 evidence: low

Strength of the 2025 recommendation: strong

What priority do you rate this topic for conversion to a living format?

◯ High priority
◯ Medium priority
◯ Low priority

Please rank the following topic:

GLUCOCORTICOIDS

Research question: In infants presenting to hospital or hospitalised with bronchiolitis, with a positive response to beta2 agonists, does administration of systemic or local glucocorticoids (nebulisation, oral, IM, or IV) improve clinically relevant end-points?

2025 recommendation: Do not use glucocorticoids for the routine treatment of infants with bronchiolitis with a positive response to beta2 agonists or other markers of a latter asthmatic phenotype outside of a RCT. Beta2 agonists should not be used in infants aged < 12 months (see R8a,b).

Quality of the 2025 evidence: not applicable (no eligible evidence)

Strength of the 2025 recommendation: strong

What priority do you rate this topic for conversion to a living format?

◯ High priority
◯ Medium priority
◯ Low priority

12. HYPERTONIC SALINE

Please select whether you think that this guideline topic is of high, medium, or low priority for conversion to a living format.

Guidance for prioritising topics for conversion to a living format:

High priority: A rating of high priority indicates that you think it is important for clinical decision-making that new evidence be rapidly incorporated to the recommendation AND you expect new evidence to emerge that would likely change the recommendation (strength, direction, or content).

Medium priority: A medium priority rating would indicate that you expect new evidence that could change the recommendation (strength, direction, or content), but the topic is less important relative to others.

Low priority: A low priority rating would indicate that the topic is less important relative to others AND you would not expect new evidence to emerge that would change the recommendation.

Please rank the following topic:

HYPERTONIC SALINE

Research question: In infants presenting to hospital or hospitalised with bronchiolitis, does administration of nebulised hypertonic saline improve clinically relevant end-points?

2025 recommendation: Do not routinely use nebulised hypertonic saline in infants presenting to hospital or hospitalised with bronchiolitis outside of a RCT.

Quality of the 2025 evidence: low

Strength of the 2025 recommendation: weak

What priority do you rate this topic for conversion to a living format?

- ◯ High priority
- ◯ Medium priority
- ◯ Low priority

13. AZITHROMYCIN

Please select whether you think that this guideline topic is of high, medium, or low priority for conversion to a living format.

Guidance for prioritising topics for conversion to a living format:

High priority: A rating of high priority indicates that you think it is important for clinical decision-making that new evidence be rapidly incorporated to the recommendation AND you expect new evidence to emerge that would likely change the recommendation (strength, direction, or content).

Medium priority: A medium priority rating would indicate that you expect new evidence that could change the recommendation (strength, direction, or content), but the topic is less important relative to others.

Low priority: A low priority rating would indicate that the topic is less important relative to others AND you would not expect new evidence to emerge that would change the recommendation.

Please rank the following topic:

AZITHROMYCIN

Research question: In infants presenting to hospital or hospitalised with bronchiolitis, does the use of azithromycin medication improve clinically relevant end-points?

2025 recommendation: Do not routinely use azithromycin for treatment of bronchiolitis in infants admitted to hospital.

Quality of the 2025 evidence: low

Strength of the 2025 recommendation: weak

What priority do you rate this topic for conversion to a living format?

- ◯ High priority
- ◯ Medium priority
- ◯ Low priority

14. ANTIBIOTIC MEDICATION

Please select whether you think that this guideline topic is of high, medium, or low priority for conversion to a living format.

Guidance for prioritising topics for conversion to a living format:

High priority: A rating of high priority indicates that you think it is important for clinical decision-making that new evidence be rapidly incorporated to the recommendation AND you expect new evidence to emerge that would likely change the recommendation (strength, direction, or content).

Medium priority: A medium priority rating would indicate that you expect new evidence that could change the recommendation (strength, direction, or content), but the topic is less important relative to others.

Low priority: A low priority rating would indicate that the topic is less important relative to others AND you would not expect new evidence to emerge that would change the recommendation.

Please rank the following topic:

ANTIBIOTIC MEDICATION

Research question: In infants presenting to hospital or hospitalised with bronchiolitis, does the use of antibiotic medication improve clinically relevant end-points?

2025 recommendation: Do not routinely use antibiotics for the treatment of infants with bronchiolitis.

Quality of the 2025 evidence: very low

Strength of the 2025 recommendation: conditional

What priority do you rate this topic for conversion to a living format?

- ◯ High priority
- ◯ Medium priority
- ◯ Low priority

Please rank the following topic:

ANTIBIOTIC MEDICATION

Research question: In infants presenting to hospital or hospitalised with bronchiolitis, does the use of antibiotic medication in infants who are at risk of developing bronchiectasis improve clinically relevant end-points?

2025 recommendation: Do not routinely use antibiotics for the treatment of bronchiolitis in infants who are at risk of developing bronchiectasis (due to known risk factors such as virus type (e.g., Adenovirus), Indigenous ethnicity, socioeconomic disadvantage).

Quality of the 2025 evidence: very low

Strength of the 2025 recommendation: weak

What priority do you rate this topic for conversion to a living format?

- ◯ High priority
- ◯ Medium priority
- ◯ Low priority

15. SUPPLEMENTAL OXYGEN AND SATURATION TARGETS

Please select whether you think that this guideline topic is of high, medium, or low priority for conversion to a living format.

Guidance for prioritising topics for conversion to a living format:

High priority: A rating of high priority indicates that you think it is important for clinical decision-making that new evidence be rapidly incorporated to the recommendation AND you expect new evidence to emerge that would likely change the recommendation (strength, direction, or content).

Medium priority: A medium priority rating would indicate that you expect new evidence that could change the recommendation (strength, direction, or content), but the topic is less important relative to others.

Low priority: A low priority rating would indicate that the topic is less important relative to others AND you would not expect new evidence to emerge that would change the recommendation.

Please rank the following topic:

SUPPLEMENTAL OXYGEN AND SATURATION TARGETS

Research question: In infants presenting to hospital or hospitalised with bronchiolitis, does administration of supplemental oxygen improve clinically relevant end-points?

2025 recommendation: Consider the use of supplemental oxygen in the treatment of hypoxaemic* infants with bronchiolitis.

*For definitions of hypoxaemic and target oxygen saturation levels, see R12b ('Oxygen saturation targets').

Quality of the 2025 evidence: low

Strength of the 2025 recommendation: conditional

What priority do you rate this topic for conversion to a living format?

- ◯ High priority
- ◯ Medium priority
- ◯ Low priority

Please rank the following topic:

SUPPLEMENTAL OXYGEN AND SATURATION TARGETS

Research question: In infants presenting to hospital or hospitalised with bronchiolitis, what level of oxygen saturation should lead to commencement or discontinuation of supplemental oxygen to improve clinically relevant end-points?

2025 recommendation: Consider the use of supplemental oxygen in infants with bronchiolitis if their oxygen saturation is:

Persistently < 90%, for infants aged ≥6 weeks; Persistently < 92%, for infants aged < 6 weeks, or infants aged < 12 months with an underlying health condition. Quality of the 2025 evidence: low

Strength of the 2025 recommendation: weak

What priority do you rate this topic for conversion to a living format?

- ◯ High priority
- ◯ Medium priority
- ◯ Low priority

16. CONTINUOUS PULSE OXIMETRY

Please select whether you think that this guideline topic is of high, medium, or low priority for conversion to a living format.

Guidance for prioritising topics for conversion to a living format:

High priority: A rating of high priority indicates that you think it is important for clinical decision-making that new evidence be rapidly incorporated to the recommendation AND you expect new evidence to emerge that would likely change the recommendation (strength, direction, or content).

Medium priority: A medium priority rating would indicate that you expect new evidence that could change the recommendation (strength, direction, or content), but the topic is less important relative to others.

Low priority: A low priority rating would indicate that the topic is less important relative to others AND you would not expect new evidence to emerge that would change the recommendation.

Please rank the following topic:

CONTINUOUS PULSE OXIMETRY

Research question: In infants hospitalised with bronchiolitis, does continuous monitoring of pulse oximetry beneficially change medical management or clinically relevant end-points?

2025 recommendation: Do not routinely use continuous pulse oximetry for medical management of non-hypoxaemic infants ($SpO_2 \geq 90\%$ for infants ≥6 weeks age, or $SpO_2 \geq 92\%$ for infants < 6 weeks age, or infants aged < 12 months an underlying health condition), with bronchiolitis not receiving oxygen, or stable infants receiving low-flow oxygen, who are not at risk of apnoea.

Quality of the 2025 evidence: moderate

Strength of the 2025 recommendation: conditional

What priority do you rate this topic for conversion to a living format?

- ◯ High priority
- ◯ Medium priority
- ◯ Low priority

17. HIGH-FLOW THERAPY

Please select whether you think that this guideline topic is of high, medium, or low priority for conversion to a living format.

Guidance for prioritising topics for conversion to a living format:

High priority: A rating of high priority indicates that you think it is important for clinical decision-making that new evidence be rapidly incorporated to the recommendation AND you expect new evidence to emerge that would likely change the recommendation (strength, direction, or content).

Medium priority: A medium priority rating would indicate that you expect new evidence that could change the recommendation (strength, direction, or content), but the topic is less important relative to others.

Low priority: A low priority rating would indicate that the topic is less important relative to others AND you would not expect new evidence to emerge that would change the recommendation.

Please rank the following topic:

HIGH-FLOW THERAPY

Research question: In infants hospitalised with bronchiolitis, does the use of high-flow nasal cannula improve clinically relevant end-points?

2025 recommendation: i. Do not routinely use HF therapy in infants with mild or moderate bronchiolitis who are not hypoxaemic.*

ii. Do not routinely use HF therapy as a first-line therapy in infants with moderate bronchiolitis who are hypoxaemic.*

iii. Consider HF therapy in infants with bronchiolitis who are hypoxaemic,* and who have failed low flow oxygen.

iv. Consider HF therapy in infants with bronchiolitis with severe disease prior to CPAP.

* For otherwise healthy infants aged ≥6 weeks: SpO_2 persistently < 90%. For infants aged < 6 weeks, or infants aged < 12 months with an underlying health condition: SpO_2 persistently < 92%.

Quality of the 2025 evidence: low

Strength of the 2025 recommendation: conditional

What priority do you rate this topic for conversion to a living format?

- ◯ High priority
- ◯ Medium priority
- ◯ Low priority

18. CONTINUOUS POSITIVE AIRWAY PRESSURE (CPAP)

Please select whether you think that this guideline topic is of high, medium, or low priority for conversion to a living format.

Guidance for prioritising topics for conversion to a living format:

High priority: A rating of high priority indicates that you think it is important for clinical decision-making that new evidence be rapidly incorporated to the recommendation AND you expect new evidence to emerge that would likely change the recommendation (strength, direction, or content).

Medium priority: A medium priority rating would indicate that you expect new evidence that could change the recommendation (strength, direction, or content), but the topic is less important relative to others.

Low priority: A low priority rating would indicate that the topic is less important relative to others AND you would not expect new evidence to emerge that would change the recommendation.

Please rank the following topic:

CONTINUOUS POSITIVE AIRWAY PRESSURE (CPAP)

Research question: In infants hospitalised with bronchiolitis, does the use of CPAP improve clinically relevant end-points?

2025 recommendation: Consider using CPAP therapy in infants with bronchiolitis and impending or severe respiratory failure, and/or with severe illness.

Quality of the 2025 evidence: very low

Strength of the 2025 recommendation: conditional

What priority do you rate this topic for conversion to a living format?	◯ High priority<br>◯ Medium priority<br>◯ Low priority

19. FEEDING DURING HIGH-FLOW THERAPY

Please select whether you think that this guideline topic is of high, medium, or low priority for conversion to a living format.

Guidance for prioritising topics for conversion to a living format:

High priority: A rating of high priority indicates that you think it is important for clinical decision-making that new evidence be rapidly incorporated to the recommendation AND you expect new evidence to emerge that would likely change the recommendation (strength, direction, or content).

Medium priority: A medium priority rating would indicate that you expect new evidence that could change the recommendation (strength, direction, or content), but the topic is less important relative to others.

Low priority: A low priority rating would indicate that the topic is less important relative to others AND you would not expect new evidence to emerge that would change the recommendation.

Please rank the following topic:

FEEDING DURING HIGH-FLOW THERAPY

Research question: In infants presenting to hospital or hospitalised with bronchiolitis and managed with high-flow therapy, does the use of enteral nutrition (oral and non-oral) impact clinically relevant end-points?

2025 recommendation: i. Consider enteral feeding (NG or oral), if tolerated, in infants receiving high flow.

ii. Consider continuous NG feeding in infants receiving CPAP who are not judged at imminent risk of intubation.

Quality of the 2025 evidence: very low

Strength of the 2025 recommendation: weak/ consensus-based

What priority do you rate this topic for conversion to a living format?

- ◯ High priority
- ◯ Medium priority
- ◯ Low priority

20. NON-ORAL HYDRATION

Please select whether you think that this guideline topic is of high, medium, or low priority for conversion to a living format.

Guidance for prioritising topics for conversion to a living format:

High priority: A rating of high priority indicates that you think it is important for clinical decision-making that new evidence be rapidly incorporated to the recommendation AND you expect new evidence to emerge that would likely change the recommendation (strength, direction, or content).

Medium priority: A medium priority rating would indicate that you expect new evidence that could change the recommendation (strength, direction, or content), but the topic is less important relative to others.

Low priority: A low priority rating would indicate that the topic is less important relative to others AND you would not expect new evidence to emerge that would change the recommendation.

Please rank the following topic:

NON-ORAL HYDRATION

Research question: In infants presenting to hospital or hospitalised with bronchiolitis, does the use of non-oral hydration improve clinically relevant end-points?

2025 recommendation: Use supplemental hydration for infants with bronchiolitis who cannot maintain hydration orally.

Quality of the 2025 evidence: not applicable (no eligible evidence)

Strength of the 2025 recommendation: strong

What priority do you rate this topic for conversion to a living format?

- ◯ High priority
- ◯ Medium priority
- ◯ Low priority

Please rank the following topic:

NON-ORAL HYDRATION

Research question: In infants presenting to hospital or hospitalised with bronchiolitis, what forms of non-oral hydration improve clinically relevant end-points?

2025 recommendation: i. Use either NG or IV routes for non-oral hydration in infants admitted to hospital with bronchiolitis requiring supplemental hydration.

ii. Consider NG as the preferred first method of non-oral hydration in infants with moderate bronchiolitis requiring supplemental hydration.

iii. Consider either continuous or bolus methods of NG non-oral hydration with oral rehydration solution, breast milk, or formula in infants admitted to hospital with bronchiolitis requiring an NG.

Quality of the 2025 evidence: moderate

Strength of the 2025 recommendation: strong

What priority do you rate this topic for conversion to a living format?

- ◯ High priority
- ◯ Medium priority
- ◯ Low priority

Please rank the following topic:

NON-ORAL HYDRATION

Research question: In infants presenting to hospital or hospitalised with bronchiolitis, does limiting the volume of non-oral hydration impact on clinically relevant end-points?

2025 recommendation: Consider fluid restriction at 50-75% of recommended maintenance due to the risk of fluid overload from SiADH, and hyponatremia in bronchiolitis. Monitor for signs of overhydration.

Quality of the 2025 evidence: not applicable (no eligible evidence)

Strength of the 2025 recommendation: consensus-based

What priority do you rate this topic for conversion to a living format?	◯ High priority<br>◯ Medium priority<br>◯ Low priority

Please rank the following topic:

NON-ORAL HYDRATION

Research question: In infants presenting to hospital or hospitalised with bronchiolitis, does the type of intravenous fluid impact clinically relevant end-points?

2025 recommendation: Consider using either 0.9% sodium chloride (normal saline) with 5% glucose, or balanced fluid (e.g., Plasma-lyte 148TM or Hartmann's solution) with 5% glucose, for use as maintenance fluid in infants admitted to hospital with bronchiolitis requiring IV hydration. For younger infants aged up to 4 weeks corrected with bronchiolitis, consider 10% glucose, or monitoring of blood sugar levels if receiving 5% glucose.

Quality of the 2025 evidence: not applicable (no eligible evidence)

Strength of the 2025 recommendation: consensus-based

What priority do you rate this topic for conversion to a living format?	◯ High priority<br>◯ Medium priority<br>◯ Low priority

21. CHEST PHYSIOTHERAPY

Please select whether you think that this guideline topic is of high, medium, or low priority for conversion to a living format.

Guidance for prioritising topics for conversion to a living format:

High priority: A rating of high priority indicates that you think it is important for clinical decision-making that new evidence be rapidly incorporated to the recommendation AND you expect new evidence to emerge that would likely change the recommendation (strength, direction, or content).

Medium priority: A medium priority rating would indicate that you expect new evidence that could change the recommendation (strength, direction, or content), but the topic is less important relative to others.

Low priority: A low priority rating would indicate that the topic is less important relative to others AND you would not expect new evidence to emerge that would change the recommendation.

Please rank the following topic:

CHEST PHYSIOTHERAPY

Research question: In infants hospitalised with bronchiolitis, does chest physiotherapy improve clinically relevant end-points?

2025 recommendation: Do not routinely use chest physiotherapy in infants with bronchiolitis.

Quality of the 2025 evidence: low

Strength of the 2025 recommendation: conditional

What priority do you rate this topic for conversion to a living format?

- ◯ High priority
- ◯ Medium priority
- ◯ Low priority

22. SUCTIONING

Please select whether you think that this guideline topic is of high, medium, or low priority for conversion to a living format.

Guidance for prioritising topics for conversion to a living format:

High priority: A rating of high priority indicates that you think it is important for clinical decision-making that new evidence be rapidly incorporated to the recommendation AND you expect new evidence to emerge that would likely change the recommendation (strength, direction, or content).

Medium priority: A medium priority rating would indicate that you expect new evidence that could change the recommendation (strength, direction, or content), but the topic is less important relative to others.

Low priority: A low priority rating would indicate that the topic is less important relative to others AND you would not expect new evidence to emerge that would change the recommendation.

Please rank the following topic:

SUCTIONING

Research question: In infants hospitalised with bronchiolitis, does suctioning of the nose or nasopharynx improve clinically relevant end-points?

2025 recommendation: i. Do not routinely use nasal suction in the management of infants with bronchiolitis.

ii. Consider using superficial suctioning in infants who have respiratory distress or feeding difficulties due to upper airway secretions.

Quality of the 2025 evidence: low

Strength of the 2025 recommendation: conditional

What priority do you rate this topic for conversion to a living format?

- ◯ High priority
- ◯ Medium priority
- ◯ Low priority

Please rank the following topic:

SUCTIONING

Research question: In infants hospitalised with bronchiolitis, does deep suctioning in comparison to superficial suctioning beneficially improve clinically relevant end-points?

2025 recommendation: Do not routinely use deep nasal suctioning for the management of infants with bronchiolitis.

Quality of the 2025 evidence: low

Strength of the 2025 recommendation: weak

What priority do you rate this topic for conversion to a living format?

- ◯ High priority
- ◯ Medium priority
- ◯ Low priority

23. NASAL SALINE

Please select whether you think that this guideline topic is of high, medium, or low priority for conversion to a living format.

Guidance for prioritising topics for conversion to a living format:

High priority: A rating of high priority indicates that you think it is important for clinical decision-making that new evidence be rapidly incorporated to the recommendation AND you expect new evidence to emerge that would likely change the recommendation (strength, direction, or content).

Medium priority: A medium priority rating would indicate that you expect new evidence that could change the recommendation (strength, direction, or content), but the topic is less important relative to others.

Low priority: A low priority rating would indicate that the topic is less important relative to others AND you would not expect new evidence to emerge that would change the recommendation.

Please rank the following topic:

NASAL SALINE

Research question: In infants hospitalised with bronchiolitis, does the use of nasal saline drops improve clinically relevant end-points?

2025 recommendation: i. Do not routinely use nasal saline drops in the management of infants with bronchiolitis.

ii. Consider a trial of intermittent nasal saline drops at time of feeding in infants with reduced feeding.

Quality of the 2025 evidence: very low

Strength of the 2025 recommendation: conditional

What priority do you rate this topic for conversion to a living format?

- ◯ High priority
- ◯ Medium priority
- ◯ Low priority

24. INFECTION CONTROL PRACTICES

Please select whether you think that this guideline topic is of high, medium, or low priority for conversion to a living format.

Guidance for prioritising topics for conversion to a living format:

High priority: A rating of high priority indicates that you think it is important for clinical decision-making that new evidence be rapidly incorporated to the recommendation AND you expect new evidence to emerge that would likely change the recommendation (strength, direction, or content).

Medium priority: A medium priority rating would indicate that you expect new evidence that could change the recommendation (strength, direction, or content), but the topic is less important relative to others.

Low priority: A low priority rating would indicate that the topic is less important relative to others AND you would not expect new evidence to emerge that would change the recommendation.

Please rank the following topic:

INFECTION CONTROL PRACTICES

Research question: In infants presenting to hospital or hospitalised with bronchiolitis, do infection control practices improve clinically relevant end-points?

2025 recommendation: i. Use hand hygiene practices for the management of infants with bronchiolitis.

ii. Consider multicomponent infection control practices for the management of infants with bronchiolitis.

iii. Consider cohorting of infants admitted to inpatient wards with bronchiolitis.

Quality of the 2025 evidence: very low

Strength of the 2025 recommendation: strong

What priority do you rate this topic for conversion to a living format?

- ◯ High priority
- ◯ Medium priority
- ◯ Low priority

25. SARS-COV-2 CO-INFECTION AND TREATMENT

Please select whether you think that this guideline topic is of high, medium, or low priority for conversion to a living format.

Guidance for prioritising topics for conversion to a living format:

High priority: A rating of high priority indicates that you think it is important for clinical decision-making that new evidence be rapidly incorporated to the recommendation AND you expect new evidence to emerge that would likely change the recommendation (strength, direction, or content).

Medium priority: A medium priority rating would indicate that you expect new evidence that could change the recommendation (strength, direction, or content), but the topic is less important relative to others.

Low priority: A low priority rating would indicate that the topic is less important relative to others AND you would not expect new evidence to emerge that would change the recommendation.

Please rank the following topic:

SARS-COV-2 CO-INFECTION AND TREATMENT

Research question: In infants presenting to hospital or hospitalised with bronchiolitis, to what extent does SARS-CoV-2 virus infection, or co-infection, contribute to disease incidence or severity?

2025 recommendation: Do not routinely use SARS-CoV-2 status to stratify increased risk for deterioration in infants with bronchiolitis. SARS CoV-2 infection or co-infection does not appear to place infants at increased risk of severe outcome from bronchiolitis.

Quality of the 2025 evidence: very low

Strength of the 2025 recommendation: weak

What priority do you rate this topic for conversion to a living format?

- ◯ High priority
- ◯ Medium priority
- ◯ Low priority

Please rank the following topic:

SARS-COV-2 CO-INFECTION AND TREATMENT

Research question: In infants presenting to hospital or hospitalised with bronchiolitis, who test positive for SARS-CoV-2, does use of therapies targeting the SARS-CoV-2 virus (steriods, antivirals) improve clinically relevant end-points?

2025 recommendation: i. Consider use of dexamethasone in hypoxic patients presenting with bronchiolitis who are also positive for SARS-CoV-2 co-infection.

ii. Consider use of remdesivir in immunosuppressed infants who are also positive for SARS-CoV-2 infection.

Quality of the 2025 evidence: not applicable (no eligible evidence)

Strength of the 2025 recommendation: consensus-based

What priority do you rate this topic for conversion to a living format?

- ◯ High priority
- ◯ Medium priority
- ◯ Low priority

COMMENTS

Do you have any further feedback about which topics should be converted to a living format? Please provide these below:

____
