# Supplementary material for: Prioritising Recommendations for Conversion to Living Mode in the Australasian Bronchiolitis Guideline: A Modified Delphi Study
Source: Clin Public Health Guidel. 2026 Mar 16;3(2):e70065. doi: 10.1002/gin2.70065 (PMC13563326; doi:10.1002/gin2.70065)
Supplement: Supplementary file 3 [file GIN2-3-e70065-s003.docx]

Loveys K, Babl FE, Cotterell E, Haskell L, O’Brien S, Oakley E, Tavender EJ, Wilson CL, Alsweiler J, Craig SS, Crawford NW, Crellin D, Crone S, Duke T, George S, Lithgow A, Peacock K, Ratoni T, Smith A, Starkie R, Thomas D, Wallace A, Zhang M, Borland ML, Dalziel SR, on behalf of the PREDICT Network. Prioritising recommendations for conversion to living mode in the Australasian Bronchiolitis Guideline: a modified Delphi study. Clinical and Public Health Guidelines. 2026.

## **Appendix 1. DELPHISTAR Reporting Checklist**

From: Niederberger, M., Schifano, J., Deckert, S., Hirt, J., Homberg, A., Köberich, S., Kuhn, R., Rommel, A., Sonnberger, M. & the DEWISS network (2024). Delphi studies in social and health sciences—Recommendations for an interdisciplinary standardized reporting (DELPHISTAR). Results of a Delphi study. *PLoS ONE 19(8):* e0304651. <https://doi.org/10.1371/journal.pone.0304651>

| **Topic** | **Section** | **Item** | **Checklist Item** | **Location where item is reported** | **Exemplary wording** |
| --- | --- | --- | --- | --- | --- |
| **I**  **Title and Abstract** |  | 1 | Identification as a Delphi study in the title | Title page | “Prioritising topics for conversion to living recommendations in the Australasian Bronchiolitis Guideline: a modified Delphi study” |
|  |  | 2 | Identification as a Delphi study in the abstract | Abstract | “Members of the 2025 ABG Guideline Development Committee were invited to participate in an online, modified Delphi study.” |
|  |  | 3 | Structured abstract | Abstract | Abstract headings: Background, Method, Results, Conclusion |
| **II**  **Context** | **Formal** | 4 | Information about the sources of funding | Title page | “KL, LH and SRD’s time has been funded by Cure Kids New Zealand. FEB’s time has been part funded by a grant from the Royal Children’s Hospital Foundation (Parkville, Australia), and a National Health and Medical Research Council (NHMRC) Leadership grant (Canberra, Australia). The views of the funders have not influenced the development nor content of the research.” |
|  |  | 5 | Information about the team of authors and/or researchers (e.g., discipline, institution) | Methods > survey content and procedures > paragraph 1 | “The survey was developed and pilot tested by the guideline co-chairs (MLB, SRD), and methodologist (KL) from the Australasian Bronchiolitis Guideline 2025 update. The Guideline co-chairs are paediatric emergency medicine specialists and academics.” |
|  |  | 6 | Information about method consulting | Methods > survey content and procedures > paragraph 1 | “External statistical consultation was not sought for the methodology.” |
|  |  | 7 | Information about the project background | Introduction > paragraphs 2, 3, 4 | Refer to paper from paragraph beginning, “Despite being a very common condition, historically bronchiolitis management in hospital has been affected by practice variation, including use of ineffective therapies and investigations (2, 3).” |
|  |  | 8 | Information about the study protocol | Methods > study design > paragraph 1 | “The protocol was registered on the Open Science Framework (ID hp2kt) (16).” |
|  | **Content** | 9 | Justification of the chosen method (Delphi) to answer the research question | Introduction paragraph 5, methods > study design > paragraph 2 | “…current guidance from both the Australian Living Evidence Collaboration (ALEC), and an international working group (consisting of members of ALEC, the National Institute for Health and Care Excellence [NICE], and the United States Grading of Recommendations, Assessment, Development, and Evaluations [US GRADE] Network) recommends that expert consensus informs which topics are included in a living guideline (12, 13).”  “A Delphi approach was considered appropriate as it has been recommended for prioritising topics for living guidelines by ALEC (12), and an international working group (ALEC, NICE, US GRADE Network) (13).” |
|  |  | 10 | Aim of the Delphi study (e.g., consensus, forecasting) | Introduction paragraph 5 | “This study aimed to prioritise topics for conversion to a living format in the Australasian Bronchiolitis Guideline using a modified Delphi approach.” |
| **III**  **Method** | **Body & Integration of knowledge** | 11 | Identification and elucidation of relevant expertise, spheres of experience, and perspectives (e.g., theory, practice, affected groups, disciplines) | Method > panel selection and recruitment section > paragraph 1 | Refer to paper from paragraph beginning, “All 29 members of the GDC of the Australasian Bronchiolitis Guideline 2025 update were invited to participate in the study over email.” |
|  |  | 12 | Handling of knowledge, expertise and perspectives which are missing or have been deliberately not integrated | Not applicable (recruitment from a closed group) | NA |
|  |  | 13 | Basic definition of expert^1^ | Methods > panel selection and recruitment > paragraph 1 | “The group was comprised of clinical and academic experts specializing in areas relevant to bronchiolitis management…”  “Panellists drew on their clinical experience and knowledge of the evidence base from their recent involvement in the Guideline update.” |
|  | **Delphi variant and modifications** | 14 | Identification of the type of Delphi variant and potential modifications (e.g., classic Delphi, real-time Delphi, group Delphi) | Methods > study design> paragraph 2 | “Methods of the classic Delphi approach were used, including iterative voting, controlled feedback, and statistical group summaries.”  “Modifications to the classic Delphi method included the lack of an open-ended first voting round… Secondly, we cannot claim that panellists were fully anonymous… Thirdly, items that met the consensus threshold (≥75% agreement) during a voting round were removed from subsequent voting rounds.” |
|  |  | 15 | Justification of the Delphi variant and modifications, including during the Delphi study, if applicable | Methods > study design> paragraph 2 | “Instead, panellists ranked existing guideline topics which they had previously prioritised as important at the outset of the Australasian Bronchiolitis Guideline 2025 update, in their capacity as members of the Guideline Development Committee (GDC) (7, 8, 17).”  “…they were recruited in their capacity as members of the GDC and were therefore aware of who else was invited to participate based on group membership. However, our procedures otherwise aimed to maintain anonymity among panellists, who were not aware of who had participated nor what their responses were.” |
|  | **Sample of experts** | 16 | Selection criteria for the experts (per round, per expert group if applicable) | Methods > panel selection and recruitment > paragraph 1 | “All 29 members of the GDC of the Australasian Bronchiolitis Guideline 2025 update were invited to participate in the study over email. The group was comprised of clinical and academic experts specialising in areas relevant to bronchiolitis management… All panellists were located in Aotearoa New Zealand or Australia.” |
|  |  | 17 | Identification of the experts | Methods > panel selection and recruitment > paragraph 1 | “All 29 members of the GDC of the Australasian Bronchiolitis Guideline 2025 update were invited…” |
|  |  | 18 | Information about recruiting and any subsequent recruiting of experts | Methods > panel selection and recruitment > paragraph 1 | “All 29 members of the GDC of the Australasian Bronchiolitis Guideline 2025 update were invited to participate in the study over email.” |
|  | **Survey** | 19 | Elucidation of the content development for the questionnaire^2^ | Methods > survey content and procedures > paragraph 1 | “The survey was developed and pilot tested by the guideline co-chairs (MLB, SRD), and methodologist (KL) from the Australasian Bronchiolitis Guideline 2025 update (17).” |
|  |  | 20 | Description of the questionnaire (content and structure) | Methods > survey content and procedures > paragraphs 1, 2; Appendix 2, 3 | “The panellists were asked to rank 38 of 41 existing guideline recommendations as high, medium, or low priority for conversion to a living format, using a drop-down menu within an online survey.”  “When ranking each topic, panellists were informed of the topic’s Population, Intervention, Comparison, Outcome (PICO) question, recommendation, recommendation strength, and evidence quality from the 2025 Guideline. The definitions for each ranking category (high, medium, low) were repeated for each item to encourage reliable responding (Figure 1; Appendices 2-3). At the end of the survey, qualitative data were sought by asking panellists for further written feedback on which topics should be converted to a living format.” |
|  | **Delphi rounds** | 21 | Number of Delphi rounds | Methods > panel selection and recruitment > paragraph 2; Methods > survey content and procedures > paragraph 3 | “The survey rounds ran from 30/07/2025 to 21/08/2025 (round one), and 26/08/2025 to 16/09/2025 (round two).”  “The limit of three voting rounds was used to avoid panel fatigue and forced consensus (14).” |
|  |  | 22 | Information about the aims of the individual Delphi rounds | Methods > survey content and procedures > paragraphs 1, 3 | “The panellists were asked to rank 38 of 41 existing guideline recommendations as high, medium, or low priority for conversion to a living format, using a drop-down menu within an online survey.”  “Round two only included topics for which the consensus threshold was not reached in round one.” |
|  |  | 23 | Disclosure and justification of the criterion for discontinuation | Methods > survey content and procedures > paragraph 4 | “The criteria for discontinuation of data collection were the first to occur of: 1) at least four topics reaching the consensus threshold for conversion to a living format; or 2) three voting rounds. It was prospectively determined that four to six topics could be converted to a living format based on the Guideline’s resources. The limit of three voting rounds was used to avoid panel fatigue and forced consensus (14).” |
|  | **Feedback** | 24 | Information about what data was reported back per round | Methods > survey content and procedures > paragraph 3 | “At the outset of round two, panellists were informed which topics were included for conversion to a living format and excluded in the first round. Controlled feedback was given in the form of anonymous, aggregated summaries of the frequency and proportion of votes per ranking category for each topic in the first round, presented in bar charts with written summaries.” |
|  |  | 25 | Information on how the results of the previous Delphi round were fed back to the experts surveyed (e.g., via frequencies, mean values, measures of dispersion, listing of comments) | Methods > survey content and procedures > paragraph 3 | “Controlled feedback was given in the form of anonymous, aggregated summaries of the frequency and proportion of votes per ranking category for each topic in the first round, presented in bar charts with written summaries.” |
|  |  | 26 | Information on whether feedback was differentiated by specific groups (e.g., by field of expertise, institutional affiliation) | Methods > survey content and procedures > paragraph 3 | “All panellists received the same information.” |
|  |  | 27 | Information about how dissent and unclear results were handled | Methods > survey content and procedures > paragraph 3 | “The consensus threshold for a topic to be converted to a living format was ≥75% of votes at high and/or medium priority, or ≥75% of votes at low priority for exclusion. Round two only included topics for which the consensus threshold was not reached in round one… Panellists were asked to use the feedback to consider whether to change their vote.” |
|  | **Data analysis** | 28 | Disclosure of the quantitative and qualitative analytical strategy | Methods > data analysis > paragraphs 1, 2 | Refer to paper from paragraph beginning, “As the data were categorical, frequency counts and proportions…” |
|  |  | 29 | Definition and measurement of consensus | Methods > survey content and procedures > paragraph 3 | “The consensus threshold for a topic to be converted to a living format was ≥75% of votes at high and/or medium priority, or ≥75% of votes at low priority for exclusion.” |
|  |  | 30 | Information on group-specific analysis or weighting of experts (e.g., theory vs. practice, discipline-specific analysis) | Methods > data analysis > paragraphs 1 | “The ratings of all panellists were given equal weighting, and no subgroup analyses were performed.” |
| **IV**  **Results** | **Delphi process** | 31 | Illustration of the Delphi study (e.g., in a flow chart) | Figure 2 | “A flow chart depicts a summary of the study rounds, including attrition and final classification of topics (Figure 2).” |
|  |  | 32 | Information about special aspects during the Delphi study (e.g., deviations from the intended approach with justification) | Results > paragraph 1 | “There were no deviations from the intended approach.” |
|  |  | 33 | Number of experts per round (both invited and participating) | Results > paragraph 1; Figure 1; Table 1 | “Of the 29 experts invited to complete the survey, 25 (86%) completed the first round. Twenty of 25 (80.0%) invited experts completed the second round.” |
|  | **Results** | 34 | Presentation of the results for each Delphi round and the final results | Results > paragraphs 2, 3; Table 2 | “A summary of the topics assessed, included, and excluded at each round are presented in Table 2.” |
| **V Discussion** | **Quality of findings** | 35 | Highlighting the findings from the Delphi study | Discussion > paragraphs 2, 3, 4 | Refer to paper from paragraph beginning, “In our study, high-flow therapy, combined glucocorticoid/adrenaline therapy, oxygen saturation targets, and CXR in infants with severe bronchiolitis with requirement for HDU/ICU level care were prioritised for conversion to a living format by a diverse, local expert panel.” |
|  |  | 36 | Validity of the results (e.g., transferability of the findings) | Discussion > strengths and limitations > paragraph 2 | “The broad spread of expertise, hospital types, and geographic locations represented within the guideline panel suggest that the prioritised topics are likely important across different types of interest-holders. However, the results might not generalise to bronchiolitis guidelines in regions dissimilar to Aotearoa New Zealand and Australia, given there may be local variability in the perceived importance of different management topics or the availability of different therapies or investigations.” |
|  |  | 37 | Reliability of the results (e.g., split half, inter-rater reliability) | Discussion > strengths and limitations > paragraph 2 | “Moreover, the findings of Delphi studies in general can have low replicability due to sensitivity to the panel make-up (14). Due to the small group size, it was not possible to conduct two concurrent panels to compare convergence in the findings.” |
|  |  | 38 | Reflection on potential limitations (e.g., number of experts, response bias) | Discussion > strengths and limitations > paragraphs 2, 3 | Refer to paper from paragraph beginning, “Use of an online survey may have helped with maintaining representation…” |

^1^ “Experts” are the participants; these can be people from academia, practice, or representatives of lived experience (e.g., patients, family members).

^2^ The term “questionnaire” stands for the survey instrument regardless of whether quantitative or qualitative items are integrated or weighted
